# Supplementary material for: Introducing a hemoglobin G-Makassar variant in HSCs by in vivo base editing treats sickle cell disease in mice
Source: Mol Ther. 2024 Oct 28;32(12):4353–71. doi: 10.1016/j.ymthe.2024.10.018 (PMC11638829; doi:10.1016/j.ymthe.2024.10.018)
Supplement: Document S1. Figures S1–S9 and Table S5 [file mmc1.pdf]

YMTHE, Volume *32*

Supplemental Information

Introducing a hemoglobin G-Makassar variant in HSCs by *in vivo* base editing treats sickle cell disease in mice

Chang Li, Aphrodite Georgakopoulou, Kiriaki Paschoudi, Anna K. Anderson, Lishan Huang, Sucheol Gil, Maria Giannaki, Efthymia Vlachaki, Gregory A. Newby, David R. Liu, Evangelia Yannaki, Hans-Peter Kiem, and André Lieber

Supplemental methods

Hemoglobin HPLC. Ion-exchange HPLC was performed to measure hemoglobins in hemolysates. We used the VARIANT II TURBO System (Bio-Rad, Hercules, CA) at the Red Cell Disorders Lab of the Harborview Medical Center by following the manufacturer's instructions. Hemolysates were prepared from EDTA blood samples collected from sCD46/Townes mice by using the Wash/Diluent Solution provided for the instrument.

Hemoglobin electrophoresis by isoelectric focusing (IEF). Detection of hemoglobin variants by IEF was performed at the Red Cell Disorders Lab of the Harborview Medical Center. The RESOLVE™ Hemoglobin kit from PerkinElmer (Waltham, MA) was used. Briefly, whole blood samples were diluted in RESOLVE™ solution to release the hemoglobin proteins in red blood cells, vortexed, and spun at 14,000×g for 4 min at room temperature. 4.5 µL of supernatants was loaded onto the agarose IEF gel provided in the kit. Electrophoretic separation was performed at 10°C with a Multiphor II Electrophoresis Unit (GE Healthcare, Chicago, IL), using a power supply at a voltage limit of 1500 V and a current and power limit of 2.75 mA/cm and 1.2 W/cm gel, respectively. The gel was fixed, stained with Coomassie blue R250, destained for clear background, and air dried for imaging analysis.

Propidium iodide staining for cell cycle analysis. 2-5 × 10^5 cells were fixed by adding 200 µl of 70% ethanol, then incubated at 4°C for 30 minutes. Following a second wash with 2 ml PBS + 2% BSA and centrifugation at 300 × g for 5 minutes, the cells were stained with 0.5 ml propidium iodide (PI)/RNase solution (Immunostep, Salamanca, Spain), incubated at room temperature for 15 minutes, and analyzed by flow cytometry to assess the DNA content.

Annexin/7-AAD staining for apoptosis analysis. The PE Annexin V Apoptosis Detection Kit I from BD Pharmingen (catalog # 559763) was used. 1 × 10^5 cells were washed twice with cold PBS and resuspended in 1X Binding Buffer provided in the kit at a concentration of 1 × 10^6 cells/ml. The cells were stained with PE Annexin V (5 µl) and 7-AAD (5 µl), incubated at room temperature for 15 minutes in the dark. After incubation, 400 µl of 1X Binding Buffer was added. Flow cytometry analysis was performed within 1 hour.

Table S1. Potential off-target sites in mouse genome identified by CIRCLE-Seq. Genomic DNA from CD46/Townes naive mice was cleaved with recombinant Cas9-NRCH plus the guide sequence sgRNA_Maka for CIRCLE-Seq. A total of 2372 sites were nominated. The top 20 sites, highlighted in grey, were further analyzed by amplicon deep sequencing. Primers used for PCR and amplicon reference sequences used for analyses were listed.

Table S2. Top-scored potential off-target sites for guide sequence sgRNA_Maka in mouse genome (mm10). Candidates were computationally predicted by Cas-OFFinder and those with mismatches =<3 nucleotides were listed. The top 10 sites highlighted in grey were amplified and further analyzed by amplicon deep sequencing. The column "Overlapping with CIRCLE-Seq candidates?" shows whether those sites are overlapping with candidates nominated by CIRCLE-Seq. If yes, the corresponding ID in Table S1 was listed.

Table S3. Top 10 off-target sites in human genome identified by CIRCLE-seq and amplicon sequencing. The sites were reported previously by Newby GA et al (PMID: 34079130). The ranking were based on off-target editing rates measured by amplicon NGS.

Table S4. Top-scored off-target sites in human genome nominated by Cas-OFFinder. Candidates were computationally predicted by Cas-OFFinder and those with mismatches =<3 nucleotides were listed. The top 10 sites highlighted in grey were amplified and further analyzed by amplicon deep sequencing. The column "Overlapping with CIRCLE-Seq candidates?" shows whether those sites are overlapping with candidates nominated by CIRCLE-Seq reported previously by Newby GA et al (PMID 34079130). If yes, the corresponding ID is listed.

Table S5. Oligo and gBlock sequences used for cloning.

ID	Name	Sequence (5' to 3')	Notes
#1FR	pBS to NRCH_F	cgacggcagcgAATTCGAGCCCAAGAAGAAGAG	For cloning pBS-ABE8e-NRCH-sgHBG#2-miR and pBS-ABE8e-NRCH-sgHBG#2-miR-v2
	pBS to NRCH_R	ccagcaccttgaatttcttgctgggcaccttgtactcgtcggtgatcacggcccagcccacagagttggtgccgatggTcaggccgatgc	
#2	sgRNA_Maka	AAATGGACTATCATATGCTTACCGTAACTTGAAAGTATTTCGATTTCTTGGCTTTATATATCTTGTGGAAAGGACGAAACACCGTTCTCCACAGGAGTCAGGTGGTTTTAGAGCTAGAAATAGCAAGTTAAAATAAGGCTAGTCCGTTATCAACTTGAAAAAGTGGCACCGAGTCGGTGCTTTTTTTCCGCGGTGGGCGCGCCGGATCCTAA	For cloning pBS-ABE8e-NRCH-sgHBB-Maka-v1
#3FR	U6gRNA-F	CGTTAATTAAGGATCCGAGGGCCTATTTCCCATGATTCC	For cloning pBS-ABE8e-NRCH-sgHBB-Maka-v1 and pBS-ABE8e-NRCH-sgHBB-Maka-v2
	U6gRNA-R	AAATCGATTAGGATCCGGCGCGCCCACCGCGGAAAAAAAGCACCGAC	
#4FR	S2 to S3_F	atatctatgacatttaaataggggattacttgaac	For cloning pHCAS3-MCS-FI-PGK-mgmt
	S2 to S3_R	ttacgattaatatttaaatATATGCCCTCATCAGCCAAATC	
#5	sgRNA_Test	AAATGGACTATCATATGCTTACCGTAACTTGAAAGTATTTCGATTTCTTGGCTTTATATATCTTGTGGAAAGGACGAAACACCGTTCTCCTCAGGAGTCAGGTGGTTTTAGAGCTAGAAATAGCAAGTTAAAATAAGGCTAGTCCGTTATCAACTTGAAAAAGTGGCACCGAGTCGGTGCTTTTTTTCCGCGGTGGGCGCGCCGGATCCTAA	For cloning pBS-ABE8e-NRCH-sgHBB-Test-v1
#6FR	S5 to S7_SwaI_F	gattaagtggcatttaaatGAATTCTTTTAAAGCTTCCCCC	For cloning pHCAS7-MCS-FI-PGK-mgmt
	S5 to S7_SwaI_R	agtaatcccctatttaaatGTCATAGATATATTTGGGGAG	
#7FR	tbs to v4_F1	tttttccgcggtgggcgcgccggatcctaatcgaTTTGAGTAATTCATACA	For cloning pHCAS7-MCS-FI-PGK-mgmt
	tbs to v4_R1	actcgagatctgagtccggtagcgctagcggatctgaccCTCACGACACCTGAAATGGA	
#8FR	tbs to v4_F2	actcagatctcgagtttagcggagtggagaagagcggagccgagcctagcagagacgagaagagctacagccaccatgaaacggaca	For cloning pBS-ABE8e-sgHBG#2-miR-v2
	tbs to v4_R2	CAGGGTCAGCACGATATCTTCC	
#9	TRAP-pA gBlock	agctggctaggaattcgctgccaccatgaaccagaagcactctagcgacttcgtggtcattaaggccgtggaagatggcgtgaacgtgatcggcctgacaagaggaacagacaccaagttccaccattccgagaagctggacaagggcgaagtgatcatcgcccagtttaccgagcacaccagcgctatcaaggtgcggggcgaggccctgatccagaccgcctacggcgaaatgaaaagcgagaaaaagtgagaattcaaacccgctgatcagcagacatgataagatacattgatgagtttggacaaaccacaactagaatgcagtgaaaaaaatgctttatttgtgaaatttgtgatgctattgctttatttgtaaccattataagctgcaataaacaagttaacaacaacaattgcattcattttatgtttcaggttcagggggaggtgtgggaggttttttaaagcaagtaaaacctctacaaatgtggtaaaactagtagcggtgggctctatggcttctgaggcggaaagaaccagctggggctctagggggtatccccacgcgccctgtagcggcgcattaagcgcggcgggtgtggtggttacgcgcagcgtgaccgctacacttgccagcgccctagcgcccgctcctttcgctttcttcccttcctttctcgccacgttcgccggctttccccgtcaagctctaaatcgggggctccctttagggttccgatttagtgctttacggcacctcgaccccaaaaaacttgattagggtgatggttcacaaatgctagagcttatcgataccgtcgacctcgagggggggcccgatcgaattcctgcagcccg	Blue, TRAP; Green, SV40pA; Underlined, EcoRI
#10FR	NG163_del_F	aaaagtttctttgcgatc	For cloning pNG163-CMV-TRAP
	NG163_del_R	tagacgtttaattcgaacaattagctaaatgaattcgag	
#11FR	TRAP to NG_F	ttagctaattgttcgaaCATGAAGAATCTGCTTAGGGTTAG	For cloning pNG163-CMV-TRAP
	TRAP to NG_R	tagacgtttaattcgaaTCGATAAGCTCTAGCATTTGTG	

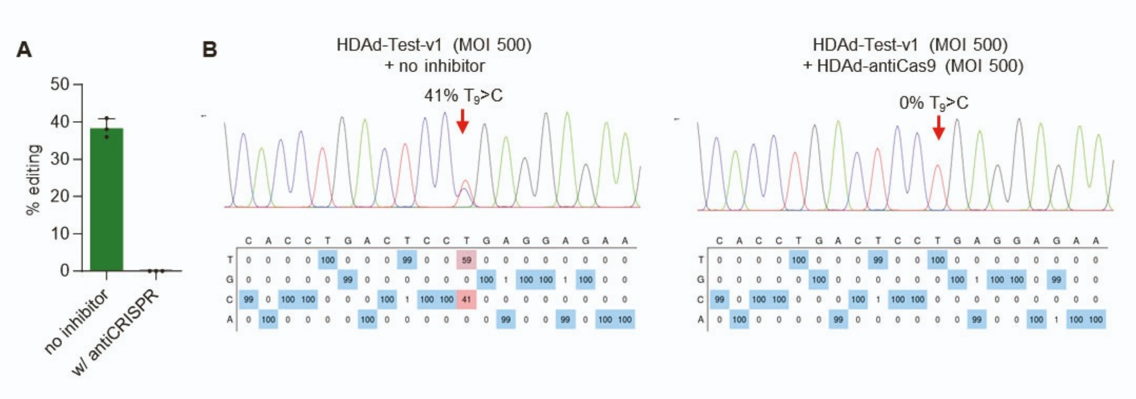

Figure S1. Blockade of base editing by anti-Cas9.

HEK293 cells were infected by HDAd-Test-v1 alone at a MOI of 500 vp/cell or together with HDAd-antiCas9 at an equal MOI. Editing was measured 4 days after vector transduction. A) Percentage of A_9 > G conversion at position 9 (T>C conversion in the complementary strand) measured by Sanger sequencing. Data shown are mean with SD of three biological replicates. B) Representative Sanger sequencing data analyzed by EditR showing base frequencies at the target region.

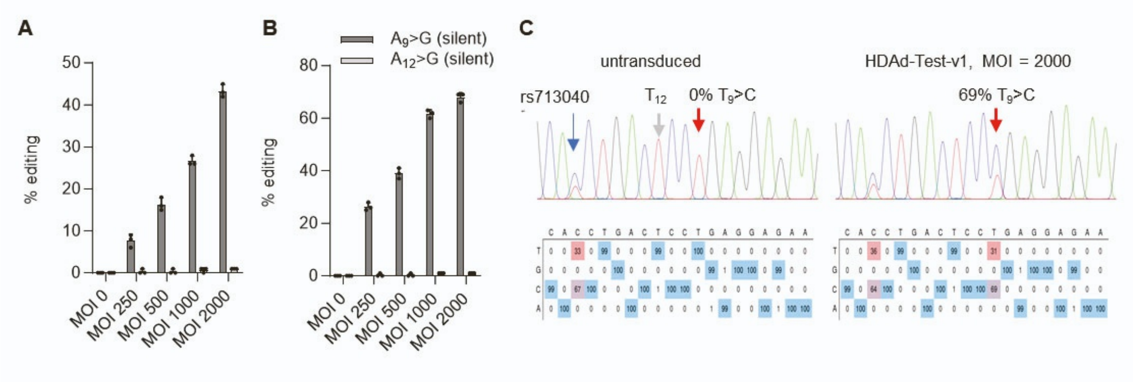

Figure S2. Editing mediated by HDAd-Test-v1.

HEK293 (**A**) or K562 (**B**) cells were infected with HDAd-Test-v1 at indicated MOIs. Base conversions at A_9 and A_{12} were measured 4 days after vector transduction by Sanger sequencing. Data shown are mean with SD of three biological replicates. **C)** Representative Sanger sequencing data of K562 cells (MOI = 2000 vp/cells) analyzed by EditR showing base frequencies at the target bases. Note that the T_9 > C conversion corresponds to A_9 > G in the complementary strand. The blue arrow points to a single-nucleotide polymorphism located in one of the three chromosome 11s of K562.

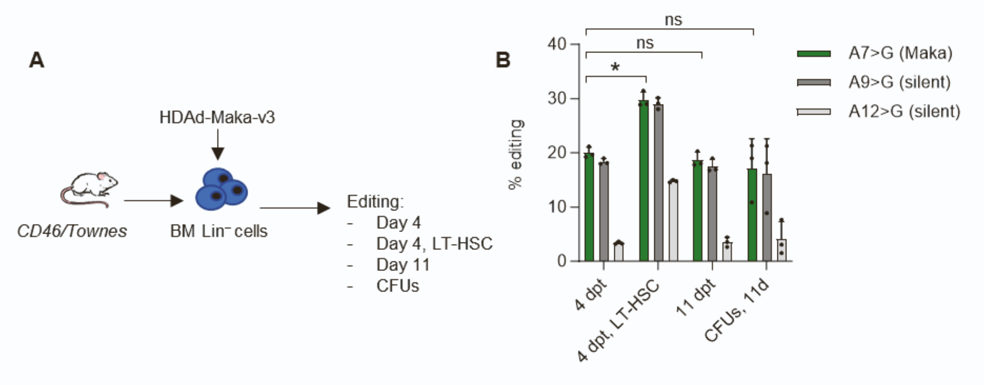

Figure S3. Editing *in vitro* after HDAd-Maka-v3 transduction and extended culture.

A) Schematic of the experiment. Bone marrow lineage-negative (BM Lin$^-$) cells from CD46/Townes mice were transduced with HDAd-Maka-v3 (MOI = 500 vp/cell). Cells were either kept in liquid culture or plated in MethoCult media for CFUs. Editing were measured in bulk BM Lin$^-$ cells or sorted long-term HSCs (LT-HSCs, CD150$^+$CD48$^-$Lin$^-$Sca-1$^+$c-Kit$^+$) at indicated time points after transduction. Pooled CFUs were harvested 10 days after plating for editing analysis. **B)** Target site editing measured by NGS. Each dot represents an individual mouse. Statistical significance was analyzed by one-way ANOVA with Šidák's multiple comparisons tests to compute p-values. dpt, days post-transduction. *, $p<0.05$; ns, not significant.

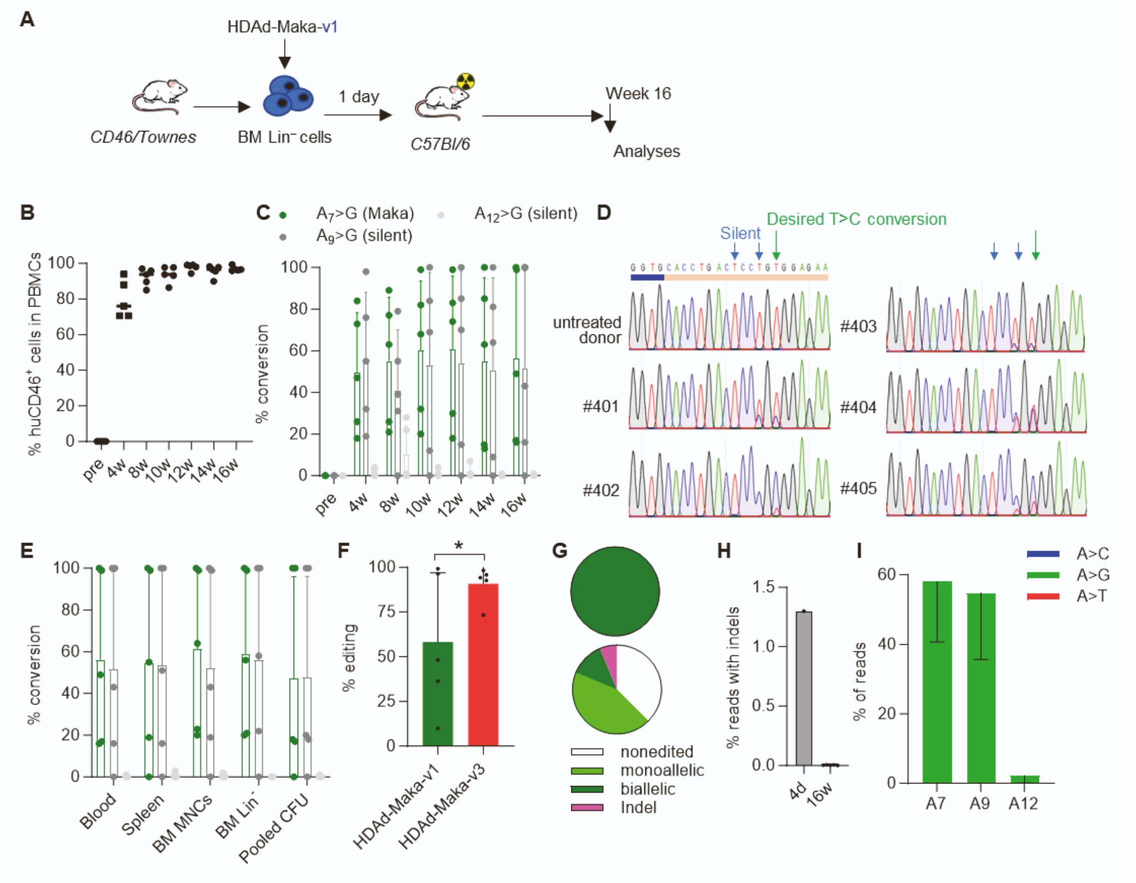

Figure S4. Test HDAd-Maka-v1 in SCD mice by *ex vivo* HSC transduction.

A) Schematic of the experiment. Bone marrow lineage-negative (BM Lin⁻) cells isolated from CD46/Townes mice were transduced with HDAd-Maka-v1 (MOI = 500 vp/cell). Cells were then either cultured for 3 days or transplanted into lethally irradiated C57Bl/6 mice 24 hours after transduction and the transplanted mice were followed for 16 weeks. **B)** Engraftment measured by flow cytometry of human CD46 expression in PBMCs. **C)** Editing in PBMCs at different time points after transplantation. A_7 > G conversion generates the Makassar variant while bystander edits at position A_9 and A_{12} are silent. **D)** Sequencing chromatograms of an untreated donor and week 4 samples after transplantation. The desired T > C conversion (A_7 > G in the complementary strand) was indicated by the green arrow. Bystander edits were indicated by blue arrows. The reference sequence is in 5' to 3' orientation. The blue-orange bar below the reference sequence illustrates the PAM-spacer mapping to the complementary strand. **E)** Editing at the target site in various tissues of primary mice at necropsy. **F)** Comparison of editing rates at the target site in week 16 primary mice. **G)** Allelic analysis in progenitor colonies derived from week 16 BM Lin⁻ cells (n=15) of primary recipients. Two mice were analyzed. **H-I)** Indels (**H**) and base substitution frequencies (I) at target site of Lin⁻ cells at necropsy measured by NGS. A > G conversions were predominant while A > C and A > T conversions were minimal. For **B, C, E, and F**, each dot represents an individual mouse.

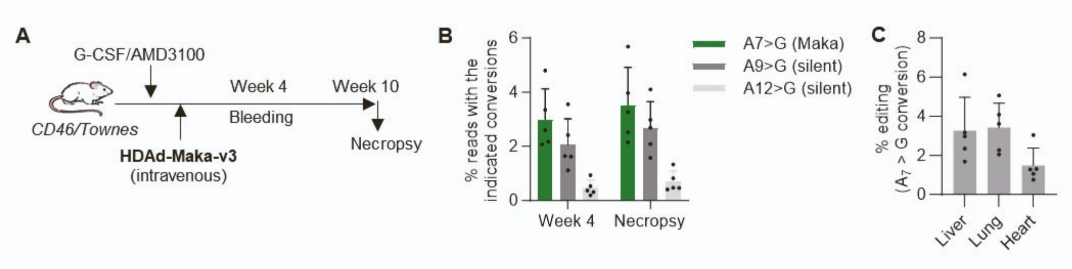

Figure S5. ***In vivo*** **HSC base editing with HDAd-Maka-v3 without** ***in vivo*** **selection.**

A) Schematic of the experiment. SCD mice (n = 5) were *in vivo* transduced with HDAd-Maka-v3 similarly as done for Figure 3. The mice were bled at week 4 and euthanized 10 weeks after in vivo transduction.

B) Editing in PBMCs (week 4) and bone marrow lineage-negative (BM Lin^{-}) cells (necropsy) were measured by NGS. **C)** Editing in tissues at necropsy measured by NGS. Each dot represents a mouse.

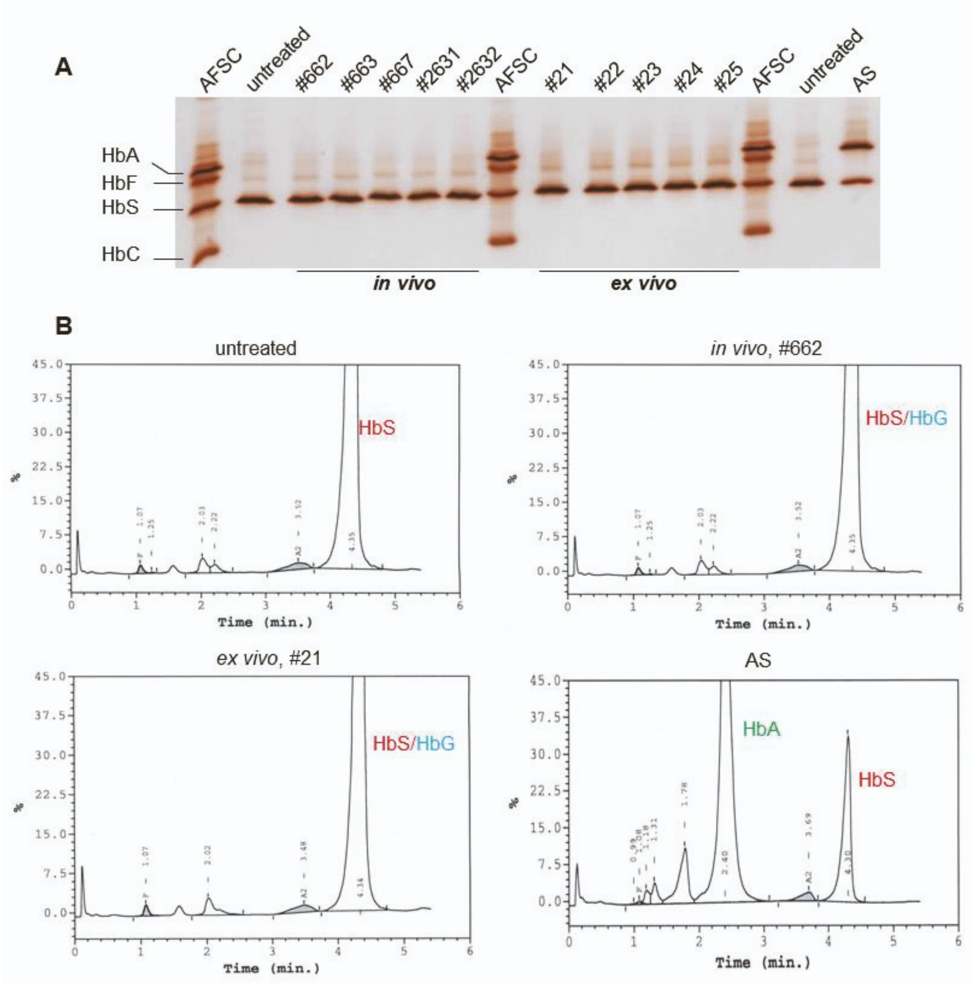

Figure S6. The G-Makassar variant overlaps with HbS in high-performance liquid chromatography (HPLC) or isoelectric focusing (IEF).

A) Analysis of hemoglobin variants by isoelectric focusing electrophoresis. Each lane represents one mouse (ear tag number labeled) or AFSC controls. Bands in AFSC controls indicating four different hemoglobin variants are labeled. **B)** Analysis of hemoglobin variants by ion-exchange high-performance liquid chromatography (HPLC). Representative chromatograms are shown. Week 16 samples of mice *in vivo* transduced with HDAd-Maka-v3 ("*in vivo*") or primary recipients transplanted with HDAd-Maka-v3-transduced CD46/Townes Lin⁻ cells ("*ex vivo*"). AS, blood sample of a control Townes mouse with the HBB^A/HBB^S genotype.

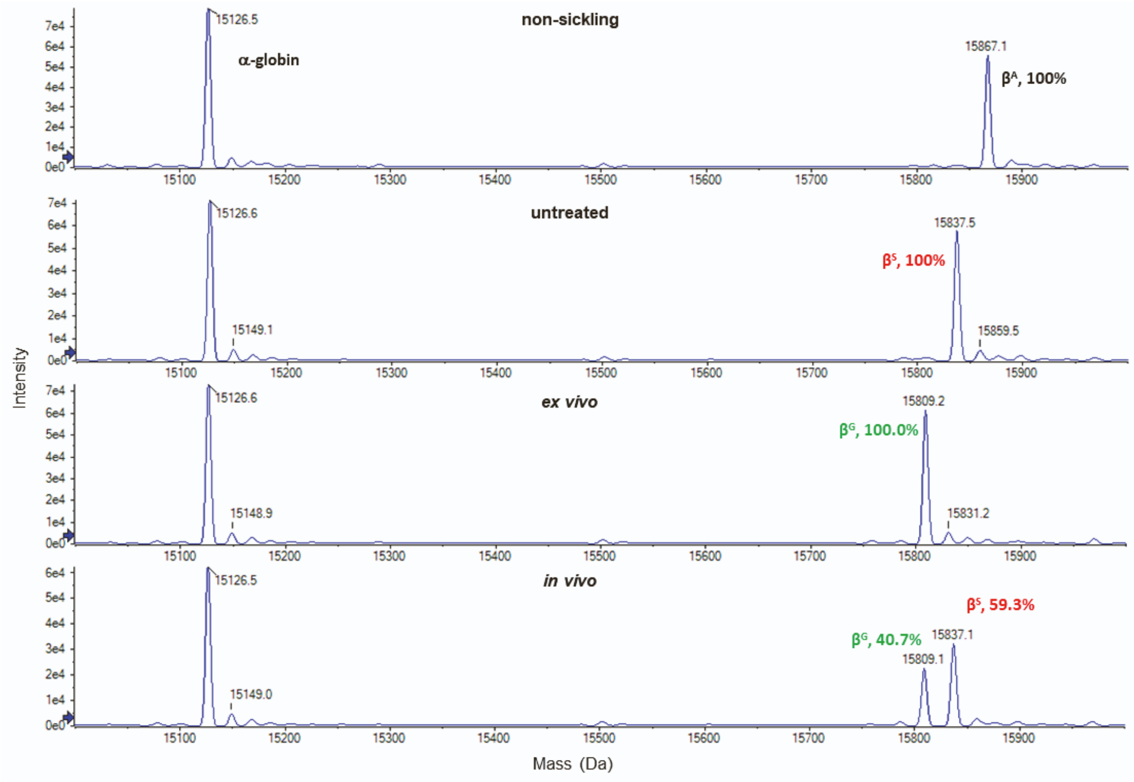

Figure S7. Complete spectrum of chromatograms showing the separation of hemoglobin subunits by mass spectrometry.

The peaks for α, β^A, β^S, β^G chains are labeled.

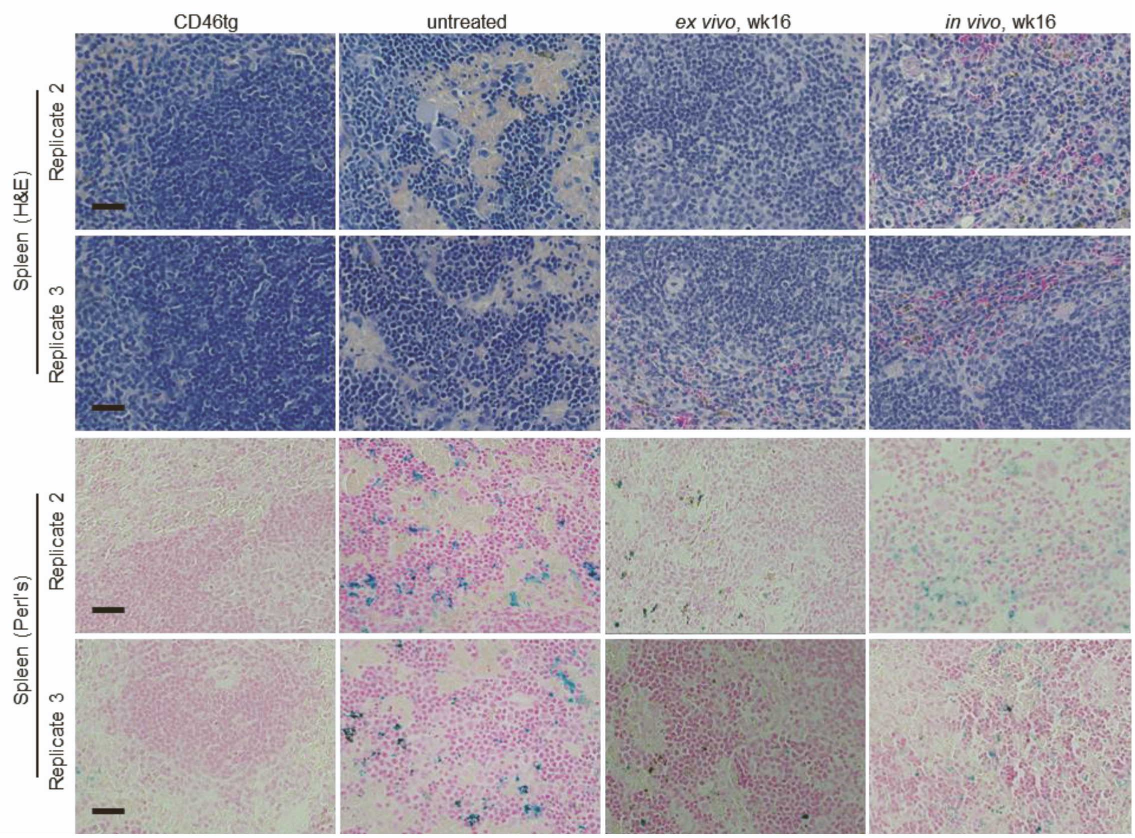

Figure S8. Two more replicates of spleen sections as shown in Figure 4F.

The sections were stained with H&E (two top panels) or Perls' Prussian blue (two bottom panels). The scale bars are 200 µm.

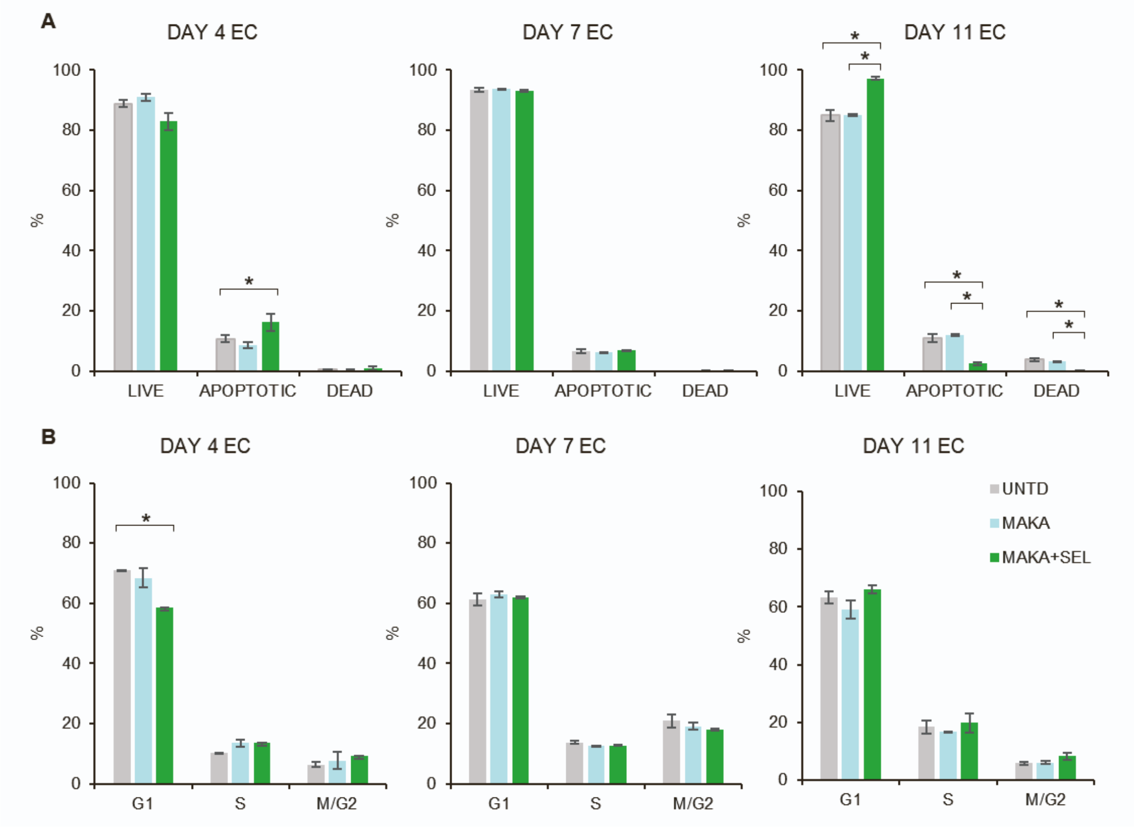

Figure S9. Cell cycle and apoptosis analyses during erythroid differentiation culture.
CD34$^+$ cells from SCD patients were treated as described in the Fig. 6 legend and cells in different stages of erythroid cells (EC) differentiation culture were analyzed. **A**) Cell cycle was measured by propidium iodide (PI) staining. **B**) Apoptosis was measured by Annexin and 7-AAD staining. Statistical significance was computed by one-way ANOVA with Šidák's multiple comparisons tests to calculate p-values. *, $p<0.05$. UNTD, untransduced. MAKA, transduced with HDAd-Maka-v3. MAKA+SEL, transduced with HDAd-Maka-v3 followed by O^6BG/BCNU selection.
