# Supplementary material for: Introducing a hemoglobin G-Makassar variant in HSCs by in vivo base editing treats sickle cell disease in mice
Source: Mol Ther. 2024 Oct 28;32(12):4353–71. doi: 10.1016/j.ymthe.2024.10.018 (PMC11638829; doi:10.1016/j.ymthe.2024.10.018)
Supplement: Document S2. Article plus supplemental information [file mmc6.pdf]

Molecular Therapy
Original Article

Introducing a hemoglobin G-Makassar variant in HSCs by *in vivo* base editing treats sickle cell disease in mice

Chang Li,[1] Aphrodite Georgakopoulou,[1] Kiriaki Paschoudi,[2] Anna K. Anderson,[1] Lishan Huang,[1] Sucheol Gil,[1] Maria Giannaki,[3] Efthymia Vlachaki,[3] Gregory A. Newby,[4,5,6,7] David R. Liu,[4,5,6] Evangelia Yannaki,[1,2] Hans-Peter Kiem,[8] and André Lieber[1,9]

[1]University of Washington, Department of Medicine, Division of Medical Genetics, Seattle, WA 98195, USA; [2]Gene and Cell Therapy Center, Hematology Department, George Papanicolaou Hospital, Thessaloniki, Greece; [3]Second Department of Internal Medicine, School of Medicine, Aristotle University of Thessaloniki, Thessaloniki, Greece; [4]Merkin Institute of Transformative Technologies in Healthcare, Broad Institute of MIT and Harvard, Cambridge, MA, USA; [5]Department of Chemistry and Chemical Biology, Harvard University, Cambridge, MA, USA; [6]Howard Hughes Medical Institute, Harvard University, Cambridge, MA, USA; [7]Johns Hopkins University, Department of Genetic Medicine, Baltimore, MD, USA; [8]Stem and Gene Therapy Program, Fred Hutchinson Cancer Research Center, Seattle, WA 98109, USA; [9]University of Washington, Department of Laboratory Medicine and Pathology, Seattle, WA 98195, USA

Precise repair of the pathogenic mutation in hematopoietic stem cells (HSCs) represents an ideal cure for patients with sickle cell disease (SCD). Here, we demonstrate correction of the SCD phenotype by converting the sickle mutation codon (GTG) into a benign G-Makassar variant (GCG) using *in vivo* base editing in HSCs. We show successful production of helper-dependent adenoviral vectors expressing an all-in-one base editor mapping to the sickle mutation site. In HSC-enriched cells from SCD patients, transduction with the base editing vector *in vitro* resulted in 35% GTG > GCG conversion and phenotypic improvements in the derived red blood cells. After *ex vivo* transduction of HSCs from an SCD mouse model and subsequent transplantation, we achieved an average of 88% editing at the target site in transplanted mice. Importantly, *in vivo* HSC base editing followed by selection generated 24.5% Makassar variant in long-term repopulating HSCs of SCD mice. The treated animals demonstrated correction of disease hallmarks without any noticeable side effects. Off-target analyses at top-scored genomic sites revealed no off-target editing. This *in vivo* approach requires a single non-integrating vector, only intravenous/subcutaneous injections, and minimal *in vivo* selection. This technically simple approach holds potential for scalable applications in resource-limiting regions where SCD is prevalent.

INTRODUCTION

Sickle cell disease (SCD) is a complex, progressive, and debilitating genetic disease caused by a single point mutation in the β-globin gene. Sickle hemoglobin (HbS) production and its subsequent polymerization causes red blood cell (RBC) sickling, which is responsible for recurrent vaso-occlusive events, chronic hemolytic anemia, and progressive vasculopathy. These clinical manifestations of SCD are associated with reduced quality of life, significant morbidity, and early mortality. A number of HSC gene therapy approaches for SCD are currently being evaluated clinically involving (1) gene addition of anti-sickling β-globin variants or fetal γ-globin, which also has powerful anti-polymerization properties,[1,2] or (2) the reactivation of fetal γ-globin by blocking repressive mechanisms mediated by the transcription factors ZBTB7A and BCL11A.[3,4] These approaches result in the formation of tetramers consisting of two α-globin chains and one or two anti-sickling globin chain(s), with β^S-globin chain largely excluded from the polymer.[1] Clinical trials have shown impressive therapeutic effects in SCD patients including reduced hemolysis and complete resolution of severe vaso-occlusive events. These approaches, however, do not remove β^S-globin. This can be achieved with prime editing that changes the sickle mutation (valine GTG) to the wild-type codon (glutamic acid GAG). We have previously reported direct repair of the sickle cell mutation *in vivo* in a mouse SCD model (CD46/Townes) using vectorized prime editors after HSC mobilization and intravenous injection of an HSC-tropic helper-dependent adenovirus (HDAd) vector.[5] This led to a cure of the disease in the CD46/Townes model. There are, however, still some potential problems with this approach. (1) When tested *in vitro* in $CD34^+$ cells from SCD patients, the target site editing efficiency with PE5 was relatively low, 5%, compared with ∼20% in CD46/Townes lineage-negative (Lin^-) cells, a bone marrow (BM) cell fraction enriched for HSCs.[5] (2) The HDAdPE5max vector transiently expresses a dominant-negative MLH1 gene to increase the efficacy and fidelity of prime editing.[6] MLH1 is involved in DNA repair, and suppressing it could increase the genotoxicity of the approach. (3) The retroviral reverse transcriptase, a part of the PE machinery, can be problematic from a safety aspect.

Received 14 June 2024; accepted 23 October 2024;
https://doi.org/10.1016/j.ymthe.2024.10.018.

Correspondence: Chang Li, University of Washington, 1705 NE Pacific Street, Seattle, WA 98195, USA.
E-mail: cli1239@uw.edu

© 2024 The Author(s). Published by Elsevier Inc. on behalf of The American Society of Gene and Cell Therapy. This is an open access article under the CC BY license (http://creativecommons.org/licenses/by/4.0/).

A

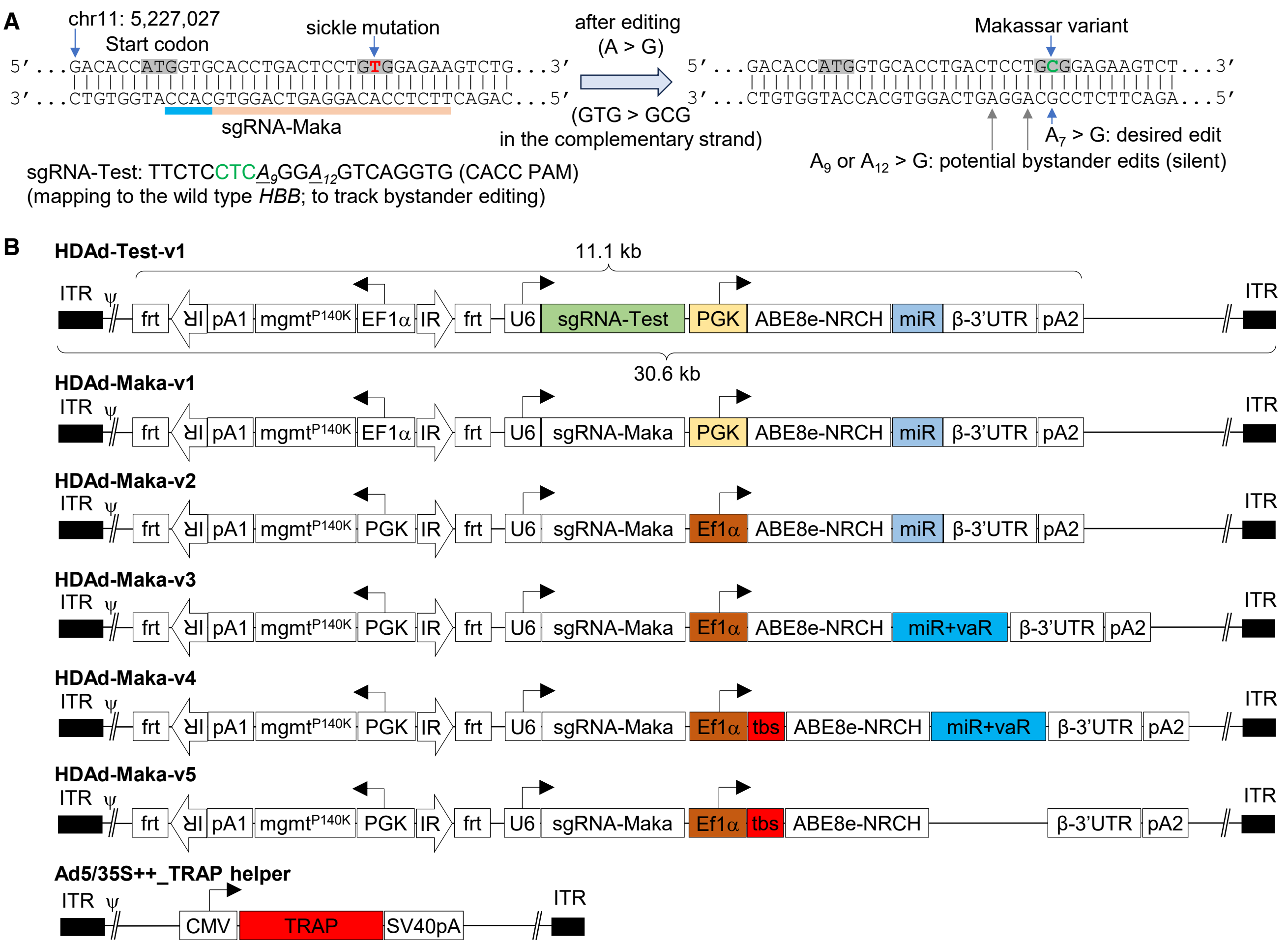

C

Name	Target sequence	Promoter of the editor	Regulation mechanism(s) of the editor	Helper virus	Rearrangements	Yield (× 10^12 vp per 3-liter spinner)
HDAd-Test-v1	*HBBA*	PGK	miR183/218	Ad5/35S++_anti-Cas9	No	2.4 ± 0.5
HDAd-Maka-v1	*HBBS*	PGK	miR183/218	Ad5/35S++_anti-Cas9	No	2.0 ± 0.4
HDAd-Maka-v2	*HBBS*	EF1α	miR183/218	Ad5/35S++_anti-Cas9	Yes	2.5 ± 0.3
HDAd-Maka-v3	*HBBS*	EF1α	miR183/218 + vaRNA	Ad5/35S++_anti-Cas9	No	1.8 ± 0.5
HDAd-Maka-v4	*HBBS*	EF1α	TRiP + miR183/218 + vaRNA	Ad5/35S++_TRAP	No	0.4 ± 0.2
HDAd-Maka-v5	*HBBS*	EF1α	TRiP	Ad5/35S++_TRAP	No	0.1 ± 0

D

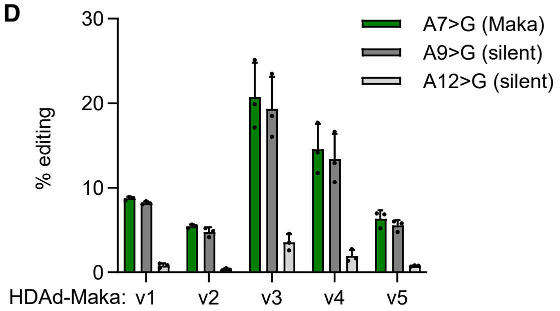

E

Allele sequence	# reads	% reads
GACACCATGGTGCACCTGACTCCTGTGGAGAAGTCTG	159947	79.9
GACACCATGGTGCACCTGACTCCCGCGGAGAAGTCTG	25175	12.6
GACACCATGGTGCACCTGACCCCCGCGGAGAAGTCTG	5042	2.5
GACACCATGGTGCACCTGACTCCTGCGGAGAAGTCTG	2575	1.3
GACACCATGGTGCACCTGACTCCCGTGGAGAAGTCTG	543	0.3
GACACCATGGTGCACCTGACTCCCGCGAAGAAGTCTG	461	0.2

(legend on next page)

A naturally occurring, non-pathogenic β-globin variant termed Hb G-Makassar at the site of the sickle mutation was first discovered in Indonesia.[7–9] Carriers of this variant exhibit normal hematologic parameters in both heterozygous and homozygous states. Therefore, conversion of the pathogenic sickle mutation to Hb G-Makassar in HSCs could represent a long-term and durable treatment strategy for SCD. This conversion would not require prime editors and could be catalyzed by modified base editors, potentially obviating the PE5-max-associated problems as outlined above. Base editors also offer significant advantages over double-strand DNA break-inducing CRISPR-Cas9, which carries risks associated with uncontrolled mixtures of insertions/deletions (indels), translocations, loss of large chromosomal segments, chromothripsis, and p53 activation.

Newby et al. generated an adenine base editor version (ABE8e-NRCH) that converts the SCD allele to the Hb G-Makassar allele with minimal non-silent bystander edits.[10,11] ABE8e-NRCH uses NRCH PAMs (R = A or G; H = A, C, or T)[12] and is therefore capable of editing the sickle 6-glutamate site. The authors achieved efficient base editing in $CD34^+$ cells from SCD patients *in vitro*, which was maintained *in vivo* after transplantation of edited cells into immunodeficient mice resulting in an ameliorated phenotype.

Here, we used ABE8e-NRCH and the sgRNA reported by Newby et al. in the context of HDAd vectors for *in vitro* and *in vivo* conversion of the sickle mutation into the Makassar variant. Notably, our *in vivo* HSC transduction approach does not require HSC transplantation. It involves the mobilization of HSCs from the BM by G-CSF/AMD3100 (Plerixafor) or other small-molecule mobilization agents such as truncated Gro-β, WU-106, and AMD3100.[13,14] While HSCs circulate at high numbers in the periphery, a single HDAd vector is injected intravenously. Transduced HSCs can return to the BM and persist long term. Edited HSCs with episomal HDAd genomes can be expanded by treatment with low-dose O^6BG/BCNU given within the first 3 weeks after *in vivo* transduction.[5,15]

We report efficient *ex vivo* conversion of the SCD mutation into the Hb G-Makassar variant in HSC-containing cell fractions from SCD patients and CD46/Townes mice. Moreover, the *in vivo* base editing of mobilized HSCs in mice resulted in a correction of the phenotype. Importantly, our *in vivo* approach requires only a single non-integrating vector and intravenous/subcutaneous injections, features that are favorable for the development of portable and affordable genetic medicines.

RESULTS

Vector design, production, and validation

We previously found that mechanisms suppressing the gene editor expression/activity specifically in vector producer (116) cells are critical for successful production of HDAd-ABE vectors.[15] We designed five HDAd vectors expressing an ABE8e-NRCH base editor for generating the Makassar variant[10] (HDAd-Maka-v1 to -v5) (Figures 1A and 1B). They differ in promoter usage (PGK in v1; EF1α in v2 to v5) and gene regulation mechanisms, including microRNA-regulated gene expression (miR183/218; v1 to v4),[15] virus-associated RNAs (vaRNAs; v3 and v4),[16] and the "transgene repression in vector production" (TRiP) system (v4 and v5).[17] The TRiP system takes advantage of bacterial tryptophan RNA-binding attenuation protein (TRAP), which recognizes its target sequence placed upstream of the start codon of the editor.[18] A helper virus containing the TRAP expression cassette was constructed and used for production for the v4 and v5 vectors (Figure 1B). For production of the other HDAd vectors, we used a helper virus expressing an anti-Cas9 gene (Ad5/35S++_Acr),[19] which almost completely blocked the base editing activity of ABE8e-NRCH (Figure S1). A vector referred to as HDAd-Test-v1 targeting the wild-type *HBB* gene (HBB^A) was constructed to track potential bystander editing at positions 9 and 12 (counting the first nucleotide at 5′ end of the spacer as position 1) in cells without the sickle cell mutation (Figures 1A–1C). The production of HDAd-Test-v1, HDAd-Maka-v1, and -v3 was successful with normal yields (2.2×10^{12} viral particles [vp] on average per

Figure 1. Design and *in vitro* validation of HDAd vectors expressing base editors

(A) Diagram showing target sequence with intended base conversions. The 20 bp guide sequence sgRNA-Maka with a CACC PAM maps to the sickle mutation site of human *HBB* gene. It is compatible with the ABE8e-NRCH editor. The desired A > G base editing at position 7 (starting from the 5′ end of the spacer) generates a GTG to GCG conversion in the complementary strand, resulting in the Makassar variant with alanine 6. Potential bystander edits at positions 9 and 12 are silent and do not affect the amino acid sequence of β-globin. The indicated sequence coordinate is based on human GRCh38.p14 primary assembly. The guide sequence for tracking bystander editing in cells with wild-type *HBB* sequence is shown on the lower left (underlined, target bases A_9 and A_{12}; green, the sixth codon of the wild-type *HBB*). The corresponding virus is named HDAd-Test-v1. (B) Schematics of HDAd vectors expressing all-in-one base editors used in this study. The overall structure of HDAd-Maka vectors contains an around 11 kb transgene element including a 2.9 kb $mgmt^{P140K}$ expression cassette flanked by two *frt*-IRs and an 8.3 kb base editor (BE) cassette. The expression of $MGMT^{P140K}$ allows for selection of transduced cells with O^6BG/BCNU. If required, the transposon can integrate into the HSC genome by SB100x transposase expressed from a second vector (HDAd-SB).[15] The BE cassette contains a guide sequence driven by a PolIII U6 promoter and mapping to the sickle mutation site of the human *HBB* gene. It also contains the ABE8e-NRCH editor under a human PGK promoter (v1) or a human EF1α promoter (v2 to v5). Different regulatory mechanisms were exploited to suppress the editor expression in 116 producer cells for efficient virus production: including microRNA responsive elements (miR, v1 to v4), virus-associated RNAs (vaRNAs or vaR, v3 and v4), and bacterial tryptophan RNA-binding attenuation protein (TRAP) binding sequence (v4 and v5). For rescuing v4 and v5 vectors, a new helper expressing TRAP is constructed. pA1, bovine growth hormone poly(A); pA2, simian virus 40 poly(A) signal; ITR, inverted terminal repeats; CMV, human cytomegalovirus promoter; ψ, packaging signal sequence. HDAd vector genomes are ∼30.6 kb. (C) Features and yields of HDAd vectors. Rearrangements were identified by restriction enzyme digestion of purified vector DNA. HBB^A, wild-type *HBB* gene; HBB^S, *HBB* with the sickle mutation; vp, viral particle. (D) Analyses of editing in CD46/Townes Lin^- cells. Transduction was performed at a MOI of 500 vp/cell. Editing was measured by next-generation sequencing (NGS) 4 days after. Each dot represents cells from a different donor mouse ($n = 3$). Error bars show standard deviations (SD). (E) Top alleles with read frequencies over 0.1%. The PAM and spacer sequences mapping to the complementary strand are highlighted in blue and orange, with the sickle mutation in the spacer shown in red. Expected base conversions are highlighted in green, while other conversions are shown in light blue. Read frequencies of edited alleles are shown on a color scale in the right column.

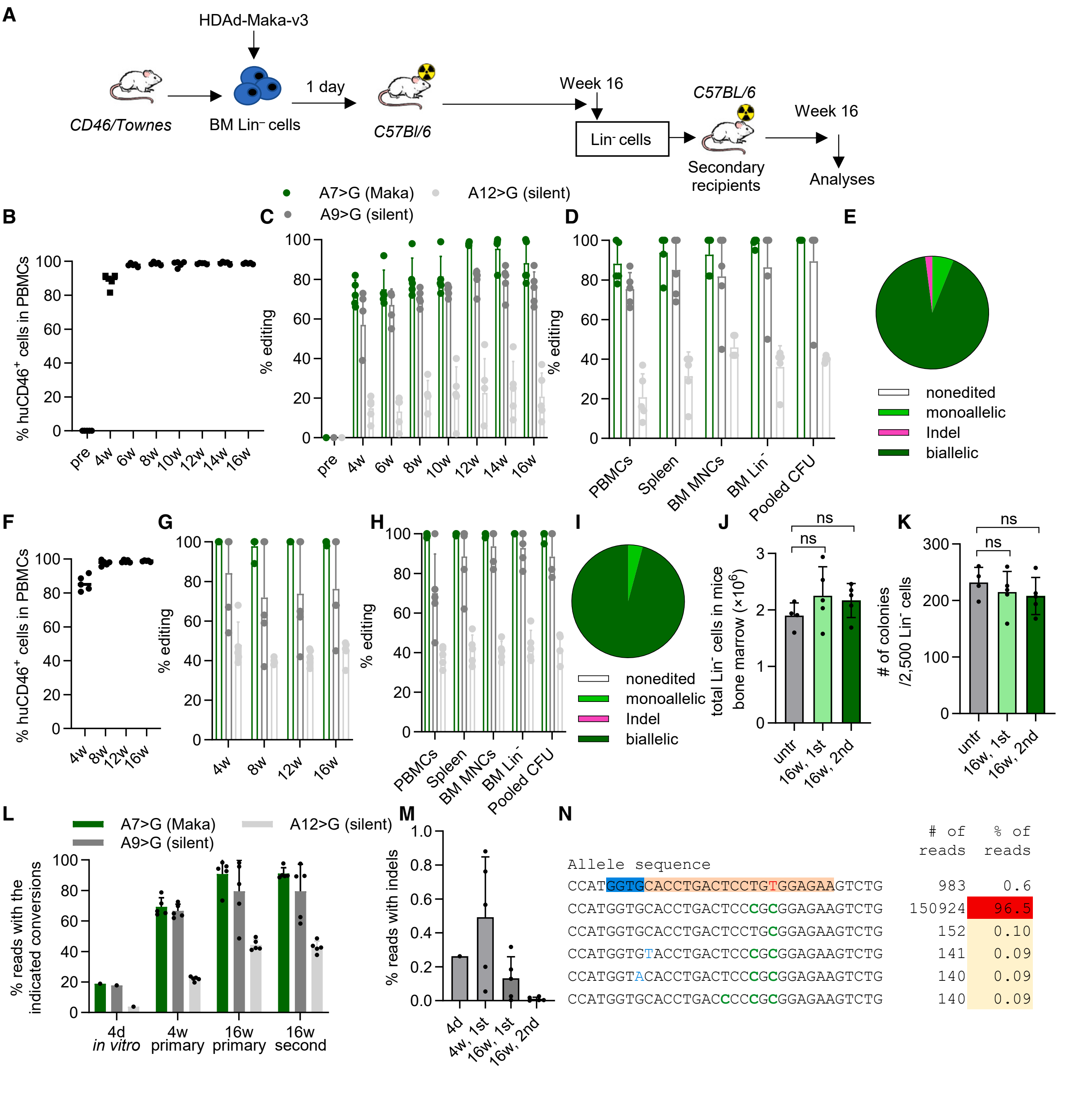

Figure 2. Generation of the Makassar variant in SCD mice by *ex vivo* HSC transduction

(A) Schematic of the experiment. Bone marrow lineage-negative (BM Lin⁻) cells were harvested from CD46/Townes mice and transduced with HDAd-Maka-v3 at an MOI of 500 vp/cell. Twenty-four hours after transduction, cells were transplanted into lethally irradiated C57Bl/6 mice. The mice were followed for 16 weeks. For further evaluation of long-term repopulating cells, BM Lin⁻ cells from these primary recipients were then used for secondary transplantation, which were monitored for another 16 weeks. (B) Engraftment of HDAd-Maka-v3-transduced HSCs in primary recipients measured by flow cytometry of human CD46 expression in PBMCs. (C) Percentages of base conversion at the target site in PBMCs at different time points after transplantation. A > G base conversion at position 7 generates the Makassar variant, while bystander edits at position A_9 and A_{12} are silent. (D) Editing at the target site in various tissues of primary mice at necropsy. (E) Allelic analysis in progenitor colonies derived from pooled week 16 BM Lin⁻ cells (*n* = 48) of primary recipients. (F–I) Analyses of secondary recipients performed similarly as for (B–E). For (C–E) and (G–I), editing was measured by Sanger sequencing. (J) Total number of Lin⁻ cells isolated from week 16 BM MNCs of primary or secondary recipients. Numbers from untreated CD46/Townes mice are shown as a comparison. (K) Number of colony-forming unit cells per 2,500 plated Lin⁻ cells. (L and M) Target base conversions (L) and indel frequencies (M) in Lin⁻ cells (week 16) of

(legend continued on next page)

3-L spinner). HDAd-Maka-v2 vector preparations were partially rearranged, likely because the microRNA-regulated gene expression alone was not sufficient to suppress the strong EF1α promoter activity. HDAd-Maka-v4 and -v5 vectors with the TRiP system were produced at low yields (0.4 and 0.1 $\times$ 10^{12} vp/spinner, respectively). We speculate this was due to a less efficient suppression of editor activity/expression by the TRiP system than the antiCas9. Future attempts to optimize the ratio of TRAP-containing helper virus to HDAd virus could potentially increase their yields. Vector features and production outcome are summarized in Figure 1C.

We validated vector preparations in cell lines and Lin^- cells from CD46/Townes mice. In cell lines without the sickle mutation, the HDAd-Test-v1 vector was used. Expected synonymous substitutions at the bystander sites were observed in HEK293 cells, with around 43% $A_9 > G$ conversion at 2,000 vp/cell. In an erythroleukemia cell line (K562), the editing level was 68% with the same vector dose (Figure S2). In Lin^- cells from SCD mice, side-by-side comparisons of the five HDAd-Maka vectors demonstrated that transduction with the HDAd-Maka-v3 vector generated the highest level of the Makassar variant. An average of 20.7% $A_7 > G$ conversion (T > C or GTG > GCG in the complementary strand) was measured 4 days after transduction (MOI = 500 vp/cell) (Figure 1D). Editing at the silent A_9 site was comparable with A_7 editing, while the $A_{12} > G$ conversion was substantially less frequent. HDAd-Maka-v1, -v2, -v4, and -v5 vectors resulted in 8.7%, 5.4%, 14.6%, and 6.3% editing, respectively, which were significantly lower than that of HDAd-Maka-v3. Over 95% of edited alleles had the desired GTG > GCG conversion (Figure 1E), demonstrating the high efficacy of ABE8e-NRCH. In consideration of the good production yield and lack of rearrangement, HDAd-Maka-v1 and HDAd-Maka-v3 were selected for downstream experiments.

Generation of the Makassar variant in SCD mice by *ex vivo* HSC transduction

Most HSC gene therapy products and clinical trials for hemoglobinopathies are based on *ex vivo* strategies. We assessed HDAd-Maka vectors in an *ex vivo* transduction setting using Lin^- cells from CD46/Townes mice. The cells were transduced with the HDAd-Maka-v3 vector followed by transplantation into lethally irradiated C57BL/6 mice (Figure 2A). Engraftment was near 100% and remained stable (Figure 2B). At week 16 after transplantation, ~88% of HBB^S alleles were converted into the G-Makassar variant HBB^G without O^6BG/BCNU selection (Figure 2C). Similar levels of $A_7 > G$ conversion at the target site were found in peripheral blood mononuclear cells (PBMCs), splenocytes, bone marrow mononuclear cells (BM MNCs), BM Lin^- cells, and pooled colony-forming units (CFU) cells derived from Lin^- cells (Figure 2D). Analyses of editing in progenitor colonies revealed that 91.8% (n = 48) were bi-allelically edited. Three colonies (6.1%) had monoallelic edits, while only one colony (2%) contained indels around the sickle mutation site (Figure 2E). Lin^- cells isolated from primary transplanted animals were able to engraft in secondary recipients (Figure 2F) with stable target site editing in peripheral blood cells (Figure 2G) and various tissues (Figure 2H), suggesting that gene modification occurred in long-term repopulating stem cells. Allelic analyses of secondary mice showed that 95.8% Lin^- cell-derived colonies had bi-allelic Makassar variants, with the remaining colonies being mono-allelically edited (Figure 2I). *Ex vivo* base editing with the HDAd-Maka-v3 vector did not alter the number of total Lin^- cells in transplanted mice and the potential of Lin^- cells to form colonies (Figures 2J and 3K). We further measured target site editing by next-generation sequencing (NGS) and found consistent frequencies of the Makassar variant, 90.8% and 91.1% in primary and secondary recipients, respectively (Figure 2L). It is notable that these editing levels are substantially higher than the 18.9% editing detected in Lin^- cells 4 days after transduction and *in vitro* culture (Figures 2L and S3). A similar expansion of editing after transplantation was also observed with other editors.[5] A contributing factor might be preferential transduction of repopulating stem cells by HDAd5/35++ vectors due to higher CD46 receptor expression on this cell subset (Figure S3).[5,20] In addition to the expected base conversions, low levels of indels were found around the nicking site. The indel frequencies were 0.13% and 0.01% on average in primary and secondary animals at week 16 (Figure 2M). It is interesting that the indel levels declined over time as 0.49% of indels were measured 4 weeks after primary engraftment. NGS data also demonstrated that an overwhelming majority of edited alleles had the installation of the Makassar mutation (GTG > GCG), while alleles with unintended base conversions around the target sites were overall below 0.1% (Figure 2N).

In addition, we tested the HDAd-Maka-v1 vector with a relatively weak PGK promoter driving the ABE8e-NRCH and showed lower targeting activity than the v3 vector *in vitro* (Figure 1D). As expected, after *ex vivo* transduction and successful engraftment (Figures S4A and S4B), lower editing frequencies with more significant variation at the target site were observed—around 50% in peripheral blood and necropsy tissues (Figures S4C–S4F). Large variation in editing was also observed by allelic analyses of Lin^- cell-derived colonies (Figure S4G), although NGS showed that the purity of edited products in Lin^- cells was similarly high, with minimal indels and rare substitutions other than the A > G conversion (Figure S4H and S4I).

Taken together, these data demonstrate that *ex vivo* transduction of Lin^- cells from SCD mice with the HDAd-Maka-v1 or -v3 vector efficiently converted the sickle cell mutation into a non-pathogenic Makassar variant without selection after transplantation into lethally irradiated recipients.

primary and secondary mice measured by NGS. For (B–D), (F–H), and (J–M), each dot represents an individual mouse. (N) Top 6 most frequent alleles in Lin^- cells (week 16) of a secondary recipient. The PAM-spacer sequence mapping to the complementary strand is indicated with the blue-orange background, with the sickle mutation in the spacer shown in red. Expected base conversions are highlighted in green, while other conversions are in light blue. Data shown are mean with SD where applicable. Statistical significance was computed by one-way ANOVA with Šidák's multiple comparisons tests to calculate p values. ns, not significant (p > 0.05).

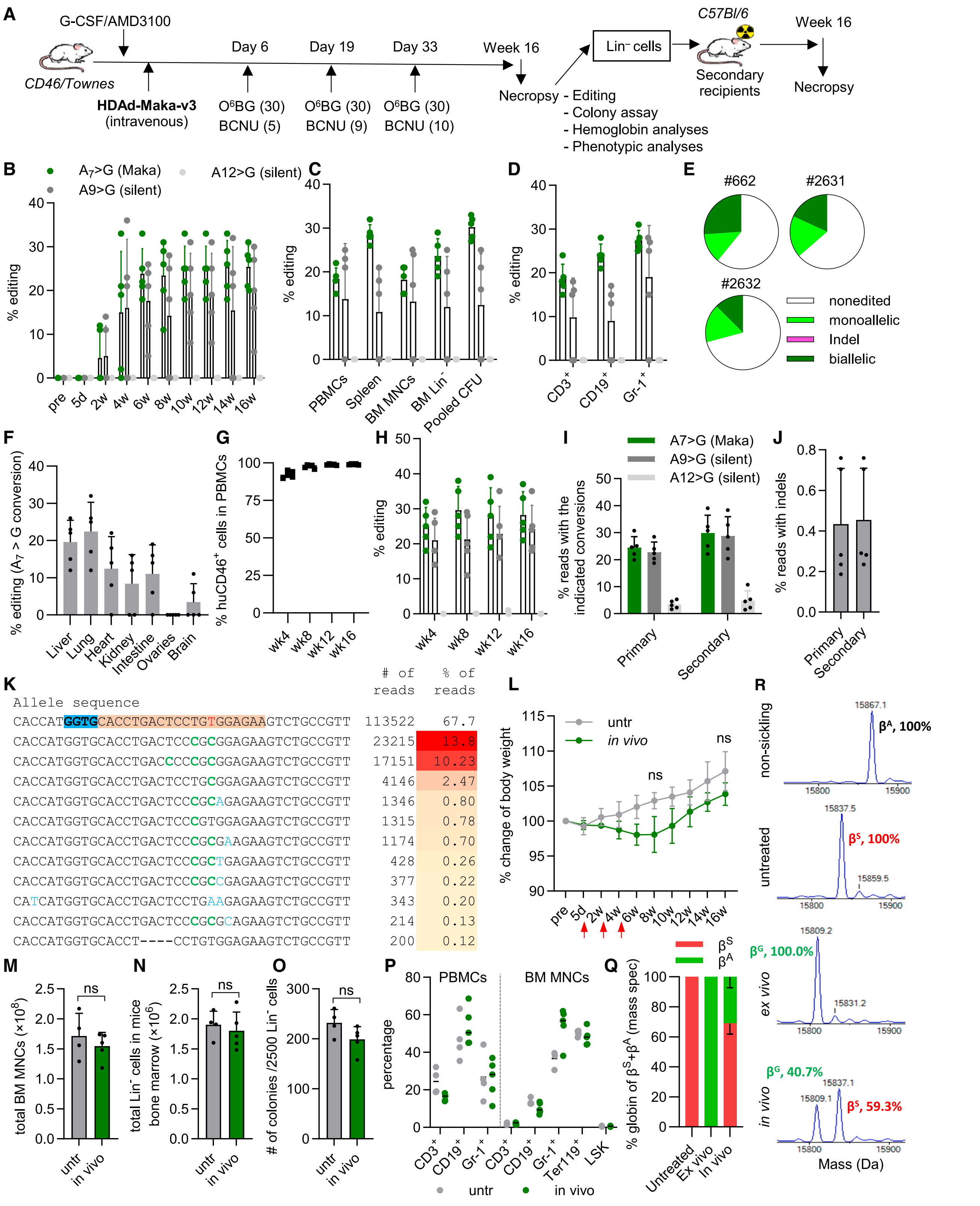

(legend on next page)

Conversion of *HBB*^{S} into a Makassar variant by *in vivo* HSC base editing

Next, we sought to generate the Makassar variant in HSCs by using a recently developed *in vivo* strategy.[20,21] HSCs of CD46/Townes mice were mobilized by G-CSF/AMD3100 and transduced *in vivo* by intravenously injecting the non-integrating HDAd-Maka-v3. To expand gene-modified cells, early selection with low doses of O^{6}BG/BCNU was started 6 days after transduction by taking advantage of the episomally expressed MGMT^{P140K}. The animals were monitored for 16 weeks followed by Lin^{-} cell isolation for secondary transplantation as performed in the *ex vivo* setting (Figure 3A). In a separate group without O^{6}BG/BCNU selection, an average of 3.0% conversion (range 2.1%–4.8%) of the sickle mutation in PBMCs and 3.5% in BM Lin^{-} cells was detected at week 10 after transduction (Figure S5). With selection, an average of 23.8% (range 18%–33%) conversion of the sickle mutation in PBMCs was achieved at week 6 and stably maintained (25.4% at week 16) (Figure 3B). Comparable levels of editing were detected in PBMCs, spleen, BM MNCs, BM Lin^{-} cells, and pooled colonies from Lin^{-} cells (Figure 3C), as well as in BM lineage cells, including CD3^{+} T cells, CD19^{+} B cells, and Gr-1^{+} myeloid cells (Figure 3D), indicating gene modification in multi-potent stem cells/progenitors. Allelic analysis of Lin^{-} cell-derived colonies showed that, on average, 34% of colonies (n = 72) had at least one edited allele with 54% of these being bi-allelically edited (Figure 3E). To investigate transduction of non-hematopoietic tissues by the HDAd5/35++ vector, we measured gene editing in various tissues 16 weeks after *in vivo* transduction. Editing in liver and lung cells were 19.6% and 22.4%. Around 10% was found in the heart, kidney, and intestine, and 3.4% was detected in brain cells (Figure 3F). This is not surprising as previous studies in transgenic mice and non-human primates with systematic delivery of HDAd5/35++ vectors showed similar vector biodistribution in these tissues.[5,20] Importantly, no editing was found in ovaries, consistent with previous findings that the reproductive system was not targeted by our vector system.[5,20] While the current study utilized female animals, future studies that include males would provide valuable evidence regarding the safety of gene targeting in male reproductive tissues following *in vivo* transduction/selection.

In secondary transplanted recipients, the engraftment of Lin^{-} cells from *in-vivo*-transduced mice was near 100% (Figure 3G). Editing rates were 29.6% at week 8 after transplantation and remained stable for the duration of the study (28.2% at week 16) (Figure 3H). This further demonstrates successful targeting of long-term repopulating cells by *in vivo* transduction. We also examined editing by NGS and observed similar results. Totals of 24.5% and 29.8% $A_7 > G$ conversion were detected in the Lin^{-} cells of *in-vivo*-transduced mice and secondary recipients, respectively (Figure 3I). Corresponding indel frequencieswere both around 0.4%, more than 50-fold lower than the desired edit (Figure 3J). When ranking allele sequences based on the number of reads, the Makassar variant was consistently detected in top-ranked and edited alleles. The silent $A_9 > G$ conversion was often introduced concurrently, whereas the synonymous substitution at A_{12} was much less frequent (Figure 3K). These patterns are similar to observations from the *ex vivo* setting (Figure 2N).

With regard to safety, no significant changes in activity and clinical presentation were observed after *in vivo* HSC transduction/selection. A transient decrease (although statistically not significant) in body weight was noted after *in vivo* selection (Figure 3L). The number of BM MNCs, Lin^{-} cells, and their ability to form colonies in semi-solid medium were not affected by the experimental procedures (Figures 3M–3O). Moreover, no changes in lineage cell composition were found in peripheral blood and bone marrow (Figure 3P).

Conversion at the protein level

We next examined the gene editing product at the hemoglobin protein level. The G-Makassar hemoglobin variant HbG differs by one amino acid from HbS. We first attempted to differentiate them at tetramer level by cation-exchange high-performance liquid chromatography (HPLC) and isoelectric focusing electrophoresis (IEF), which are used at the Harborview Medical Center in Seattle for clinical samples. However, both methods were not able to separate the two variants (Figure S6). This finding is in agreement with previous

Figure 3. *In vivo* HSC base editing with HDAd-Maka-v3 to generate the Makassar variant in SCD mice

(A) Schematic of the experiment. Mice (n = 5) were mobilized by G-CSF/AMD3100 and *in-vivo*-transduced with HDAd-Maka-v3. *In vivo* selection with O^{6}BG/BCNU was started at day 6 after HDAd injection and repeated on days 19 and 33 at the indicated doses. The mice were euthanized 16 weeks after transduction. Lin^{-} cells were isolated from bone marrow and intravenously injected into lethally irradiated C57BL/6J mice. The secondary transplanted mice were followed for another 16 weeks for specified necropsy analyses. (B–F) Editing analyses of *in-vivo*-transduced mice. (B) Target site editing in PBMCs at different time points after transduction. (C) Editing in PBMCs, spleen, BM MNCs, BM Lin^{-} cells from different tissues at necropsy. (D) Editing in lineage cells from sorted out BM MNCs. (E) Allelic analysis in progenitor colonies derived from week 16 BM Lin^{-} cells (n = 24) of three mice with indicated ear tag number. (G) Engraftment of Lin^{-} cells from *in-vivo*-transduced mice in secondary recipients. (F) Percentage of target $A_7 > G$ conversion in genomic DNA isolated from different organs at necropsy. (H) Editing in PBMCs of secondary recipients. (I–K) Target base conversions (I) and indel frequencies (J) measured by NGS. Week 16 BM Lin^{-} samples from primary and secondary mice were used. (K) Top alleles with frequencies over 0.1% in *in-vivo*-transduced mice. The PAM-spacer sequence mapping to the complementary strand is indicated with the blue-orange background, with the sickle mutation in the spacer shown in red. Expected base conversions are highlighted in green while other conversions are in light blue. (L) Body weight of untreated or *in-vivo*-transduced mice. (M) Number of BM MNCs from untreated or *in-vivo*-transduced mice. (N) Number of Lin^{-} cells isolated from week 16 BM MNCs of untreated or *in-vivo*-transduced mice. (O) Number of colony-forming unit cells per 2,500 plated Lin^{-} cells. (P) Lineage composition of PBMCs and BM MNCs of *in-vivo*-transduced mice with untreated ones as a control. (Q and R) Analyses of hemoglobin variants by mass spectrometry. Whole-blood samples at week 16 after *in vivo* and *ex vivo* (n = 5 each) transduction were analyzed. Samples from untreated CD46/Townes mice (n = 3) were used as a control. (Q) Summary of percentages of hemoglobin subunits. (R) Representative chromatogram pattern showing the separation of β^{A} from β^{S} globin chains. The labeled percentages were calculated based on the peak areas. For (B–D), (F–J), and (M–P), each dot represents one animal. Data shown are mean with SD where applicable. ns, not significant (p > 0.05 analyzed by two-tailed Student's t tests).

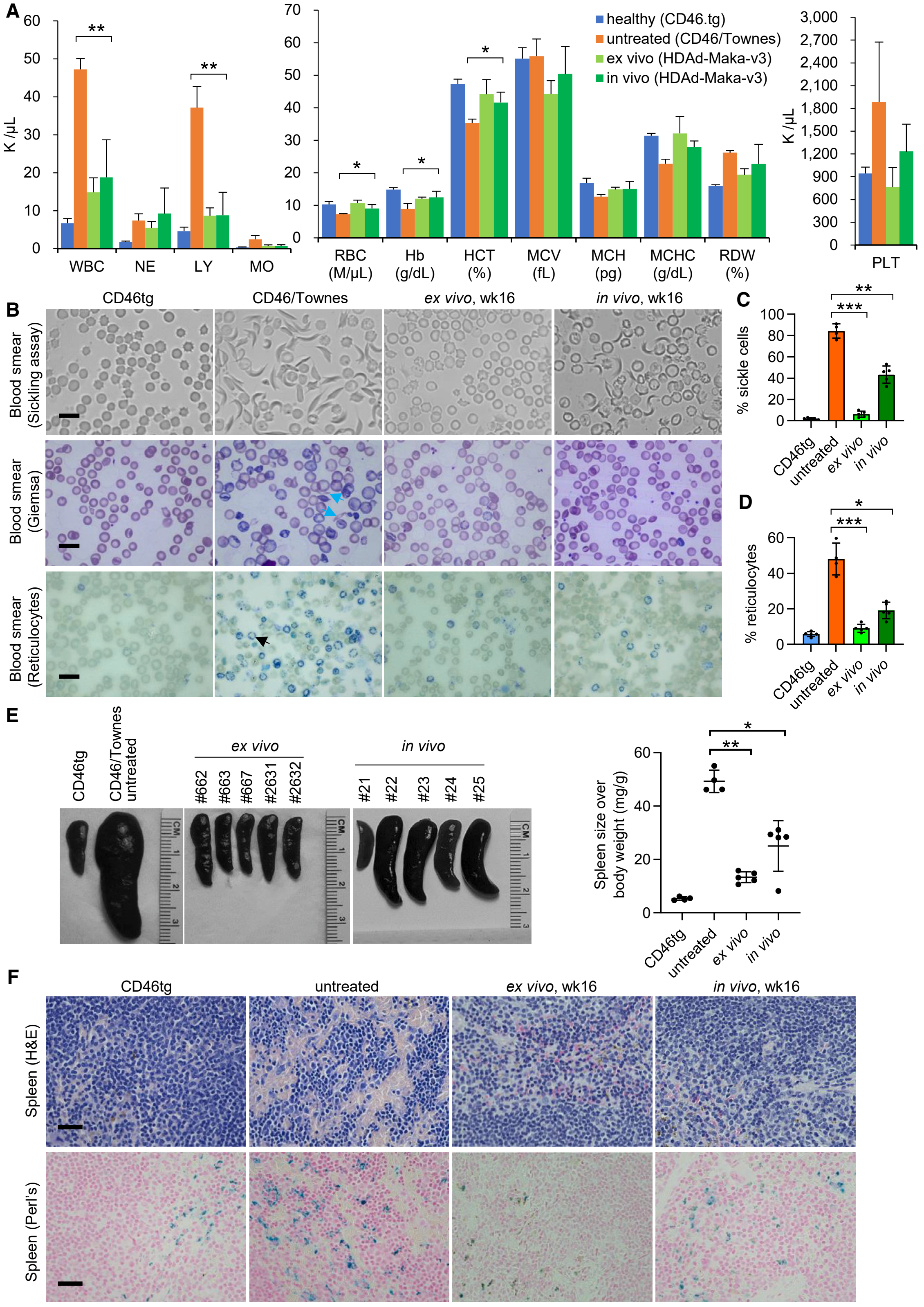

(legend on next page)

reports describing that HbS and HbG share identical properties in routine cation-exchange HPLC and IEF.[22] We therefore measured hemoglobin subunits by using mass spectrometry, which well differentiated β^G from β^S (Figure S7). In blood samples from *ex-vivo*-transduced mice, the only β-globin variant detectable was the Makassar form β^G, suggesting complete conversion of β^S into β^G (Figures 3Q and 3R). In *in-vivo*-transduced samples, on average 31.1% β^G of total β-like globin variants were found. These observations are in line with the genome editing levels described above and confirm the successful generation of the Makassar variant by precision base editing in either setting.

Phenotypic improvements after the generation of the Makassar variant

The CD46/Townes mice model resembles SCD in several key disease characteristics, such as leukocytosis, sickled RBCs, anemia, insufficient hemoglobin levels, high reticulocyte count, and splenomegaly. We assessed changes of these parameters in the treated animals. Complete blood cell counts showed that in both *ex-vivo*- and *in-vivo*-treated mice the numbers of leukocytes were close to normal counts (as seen in CD46 control mice). Significant increases in RBC counts and percentages were observed after treatment. The average hemoglobin level in naive CD46/Townes mice was 8.88 g/dL, while in *in vivo* base edited animals 12.44 g/dL was measured (Figure 4A). These data demonstrate normalization of key hematological parameters following installation of the Makassar variant.

We then evaluated RBC morphology in blood specimens. Most RBCs (84%) of CD46/Townes mice exhibited an elongated and sickled shape in an *in vitro* sickling test using sodium metabisulfite. On the contrary, no remarkable sickling was observed in blood samples from *ex-vivo*-treated animals. In samples from *in-vivo*-treated mice, a substantially decreased level (43.4%) of sickled RBCs was measured (Figures 4B and 4C). Similarly, the percentages of reticulocytes in blood smears were significantly reduced from 48.0% without treatment to 9.0% and 19.1% after *ex vivo* and *in vivo* transduction with HDAd-Maka-v3, respectively (Figures 4B and 4D).

Another hallmark of SCD is splenomegaly caused by compensatory extramedullary hemopoiesis. After conversion of the sickle mutation into the Makassar variant, we observed remarkable reduction of spleen size (Figure 4E). Further histological analyses of spleen sections revealed that base edited animals showed normalized architecture with largely regressed parenchymal iron deposition and extramedullary hemopoiesis (Figure 4F).

In summary, these data together demonstrate that generation of the Makassar variant by HDAd-Maka-v3 transduction resulted in a largely ameliorated disease phenotype in the SCD mouse model.

Analyses of off-target editing

We used both *in silico* and experimental methods to identify potential off-target sites in the mouse genome. By CIRCLE-seq,[23] a highly sensitive experimental off-target identification method based on *in vitro* cleavage of the CD46/Townes genome with Cas9-NRCH complexed with the *HBB*^{S}-targeting sgRNA and subsequent NGS, a total of 2,372 off-target candidates were nominated (Table S1). The top 20 ranked sites with the most read numbers were further amplified from the genomic DNA of Lin$^-$ cells from two untreated mice and two *in-vivo*-transduced mice with the highest on-target editing (30.8% and 25.1%). All 20 candidates are intergenic or located in introns. Amplicon NGS revealed no significant off-target base editing, i.e., A > G base conversions within the editable window (positions 3–14) (Figures 5A and 5B). By the algorithm of Cas-OFFinder,[24] 23 genomic sites with ≤3 bp mismatches were computationally predicted. Twenty-one of them are intergenic or within introns; 2 are in an exon of predicted genes. Seventeen of them were also identified by CIRCLE-seq (Table S2). Targeted sequencing of the top 10 sites showed no remarkable A > G conversions (Figures 5C and 5D). The indel frequencies around the predicted nicking sites of these candidates, mostly below 0.1%, were comparable between untreated and *in-vivo*-transduced samples. Next, we investigated off-target editing in CD34$^+$ cells from SCD patients following *in vitro* transduction with HDAd-Maka-v3. Similarly, no significant off-target activity was found in any of the top 10 CIRCLE-seq or Cas-OFFinder sites in the human genome (Figures 5F–5J; Tables S3 and S4). Notably, point mutations consistent with adenine base editing, predominantly silent or in non-coding regions, were observed by a previous report using RNP or mRNA electroporation that achieved ~80% on-target editing.[10] We hypothesize that the expression of base editors from the adenoviral genome may not reach the same high concentration that is achieved after electroporation, which underlies the improved specificity observed in this study. Taken together, our data

Figure 4. Phenotypic analyses of SCD mice after *ex vivo* and *in vivo* HSC base editing

(A) Complete blood counts from *ex-vivo*- or *in-vivo*-treated mice ($n = 5$ each). Blood samples of primary mice at necropsy were used. Untreated CD46 and Townes mice ($n = 4$ each) were analyzed as controls. WBC, white blood cells; NE, neutrophils; LY, lymphocytes; MO, monocytes; RBC, red blood cells; Hb, hemoglobin; HCT, hematocrit; MCV, mean corpuscular volume; MCH, mean corpuscular hemoglobin; MCHC, mean corpuscular hemoglobin concentration; RDW, red cell distribution width; PLT, platelets. (B) Representative microphotographs of blood cell smears. EDTA blood samples of primary mice at necropsy were used. Untreated CD46 and Townes mice were analyzed as controls. Top panel: smears of total blood cells subjected to an *in vitro* sickling assay. Middle panel: blood cell smears stained with Giemsa (blue arrows pointing to elongated RBCs). Bottom panel: staining of blood smears for reticulocytes with Brilliant cresyl blue, which stains nuclear remnants of basophilic ribonucleoproteins in reticulocytes (black arrow). Scale bars, 20 μm. (C) Percentage of sickle cells in blood smears after *in vitro* sickling assay. (D) Percentage of reticulocytes in blood smears stained with Brilliant cresyl blue. (E) Spleen sizes (left panel) and spleen weight relative to body weight (right panel). (F) Spleen sections stained with H&E (top panel) or Perls' Prussian blue (bottom panel). Iron deposition is shown as cytoplasmic blue pigments of hemosiderin in spleen tissue sections (bottom). Scale bars, 200 μm. Data shown are mean with SD where applicable. For (C) and (D), and the dot plot in (E), each symbol represents an individual mouse. Statistical significance was computed by one-way ANOVA with Šidák's multiple comparisons tests to compute p values. $^*p < 0.05$, $^{**}p < 0.01$, $^{***}p < 0.001$.

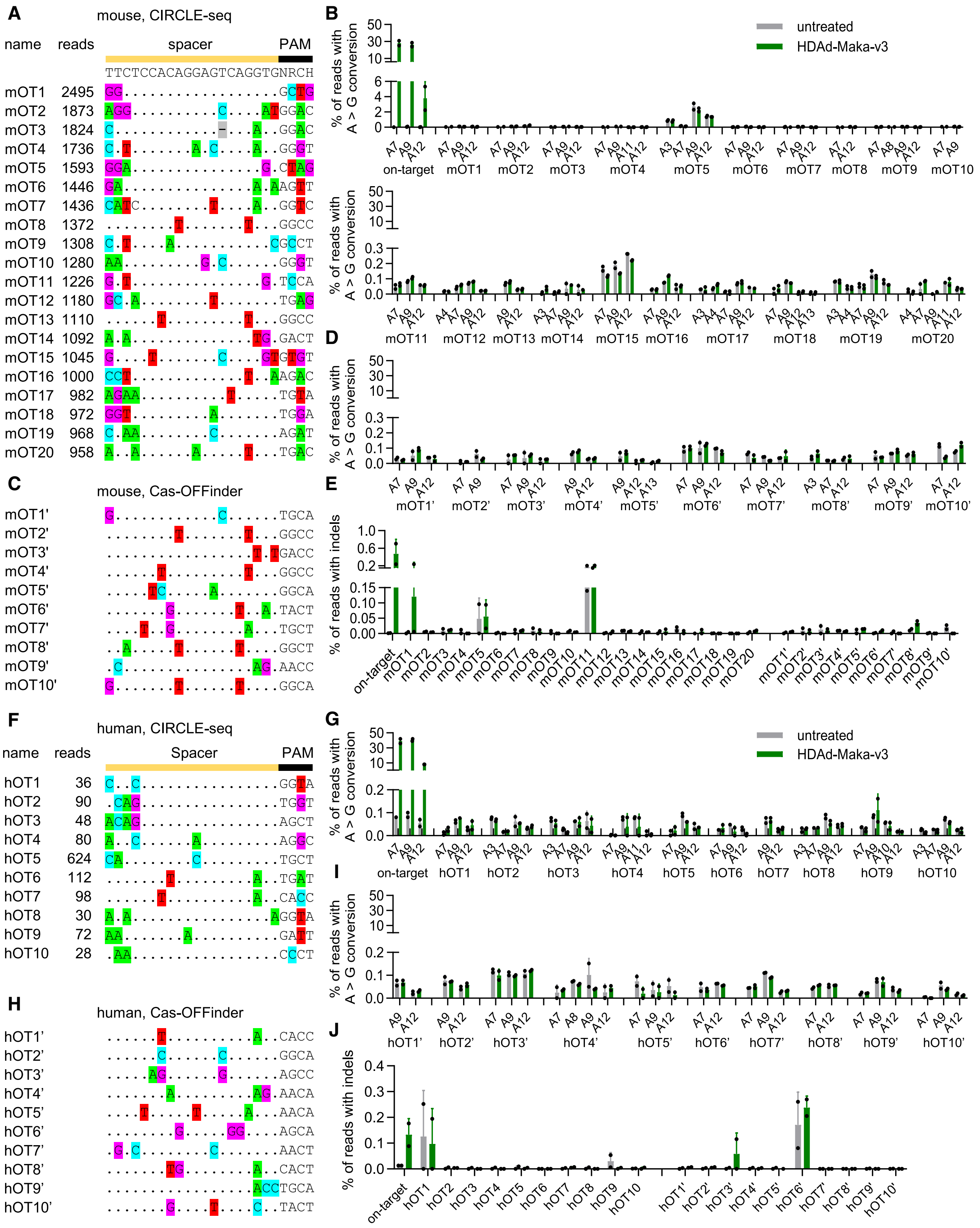

(legend on next page)

demonstrate the overall good fidelity of the vectorized ABE8e-NRCH for Makassar variant installation.

In vitro studies with $CD34^+$ cells from SCD patients

$CD34^+$ cells isolated from non-mobilized peripheral blood of three $HBB^{S/S}$ patients during exchange transfusion were subsequently transduced with the HDAd-Maka-v3. Following transduction, the cells were subjected to erythroid differentiation with or without *in vitro* selection with O^6BG/BCNU (Figure 6A). The conversion rate $A_7 > G$ reached approximately 35% in the HDAd-Maka-v3-transduced cells and near complete in the transduced and selected cells by day 11 of *in vitro* erythroid differentiation (Figures 6B and 6C). Notably, the production of G-Makassar Hb variant boosted the proliferation of transduced cells during culture (Figure 6D) and doubled the expansion rate of the transduced and selected cells compared with untransduced cells. Importantly, transduction with HDAd-Maka-v3, either alone or followed by O6BG/BCNU treatment, enhanced the clonogenic capacity of treated cells, leading to a significant increase in BFU-E and total CFU formation compared with the untransduced controls (Figure 6E). Cell-cycle and apoptosis analyses during erythroid differentiation culture (ECD) in the HDAd-Maka-v3 and selected cell group indicated increased cell proliferation and apoptosis at early time points, likely reflecting potential early toxicity from the selection process. However, at later stages of ECD, the transduced and selected cells exhibited significantly higher viability than both untransduced and the unselected transduced groups, possibly due to the enhanced survival of corrected SCD cells during differentiation (Figure S9). Moreover, the efficient generation of the G-Makassar allele significantly reduced reactivate oxygen species (ROS) (Figure 6F), a hallmark of SCD, and improved the erythroid differentiation/maturation. Specifically, transduction with the HDAd-Maka-v3, with or without O^6BG/BCNU selection, resulted in a significantly higher percentage of enucleated cells ($CD235A^+$/$NucRed^-$ cells) (Figure 6G). The observed increase in terminally differentiated cells in the transduced and selected group likely accounts for the reduced expansion rate at later ECD stages compared with earlier time points and the other cell groups (Figure 6D, day 18). Improved erythropoiesis after HDAd-Maka-v3 treatment was also demonstrated by the presence of more differentiated cells (orthochromic erythroblasts, maturing erythrocytes, reticulocytes) in the patient-derived samples during microscopic analysis (Figure 6H). In addition, while untransduced cells demonstrated robust sickling after sodium metabisulfate treatment during the sickling assay, a substantially lower percentage of sickle cells was observed in the transduced groups (Figure 6I), suggesting an effective reversal of the sickling phenotype and functional improvement.

DISCUSSION

We produced vectorized base editors for efficient conversion of the sickle cell mutation into the benign Makassar variant in patient $CD34^+$ cells and the CD46/Townes mouse model. We utilized the HDAd5/35++ vector, which efficiently transduces primitive HSCs from humans, human CD46-transgenic mice, and non-human primates.[20,25] High-level base editor expression, provided by the EF1α promoter (e.g., in HDAd-Maka-v3) appeared to be essential for efficient editing as indicated by comparative studies with the relatively weak PGK promoter.

In vitro transduction studies with HSC-enriched cell fractions from CD46/Townes (BM Lin^- cells) and SCD patients ($CD34^+$ cells) resulted in target site editing ($A_7 > G$) at a rate of $\sim$21% and 35%, respectively. Recently, we performed a similar *in vitro* editing study using a prime editing vector that mediates the correction of the SCD mutation (HDAd-PE5max).[5] Editing rates in CD46/Townes Lin^- cells and patient $CD34^+$ cells were on average 20% and 4.5%, respectively, indicating lower efficiency of the prime editor in human $CD34^+$ cells. In this study, editing frequencies in cells derived from $HBB^{S/S}$ $CD34^+$ cells and subjected to erythroid differentiation increased to over 90% after one round of treatment with O^6BG/BCNU. As a result, *in vitro* hallmarks of SCD, such as elevated ROS levels, disturbed/inefficient erythroid differentiation, and erythrocyte sickling were greatly improved.

In an *ex vivo* setting, BM Lin^- cells from CD46/Townes mice were transduced with HDAd-Maka-v3 and subsequently transplanted into irradiated C57Bl/6 mice followed by a secondary transplantation. Importantly, no O^6BG/BCNU selection was performed in this *ex vivo* study. Target site editing levels were 88% with >95% bi-allelic edits and less than <0.5% indels. The increase from 21% in the transplanted cells to 88% (at the end of week 16 after transplantation in secondary mice) likely indicates the preferential transduction of long-term repopulating HSCs or the preferential expansion of erythroid progenitors due to a near complete HbS to HbA conversion and the absence of HbS. Notably, this level of efficacy has yet to be seen in the context of ongoing lentivirus vector-based *ex vivo* HSC gene therapy trials for SCD. In studies with the currently approved SCD products CASGEVY (exagamglogene autotemcel, Vertex Pharmaceuticals and CRISPR Therapeutics)[26] and LYFGENIA (lovotibeglogene autotemcel, BlueBird Bio),[27] expression of the anti-sickling β-globin was found in 45% of erythrocytes. Our vectorized base editor also presents advantage in low-cost production and scalability over previous *ex vivo* mouse studies using RNP or mRNA electroporation to transfect

Figure 5. Analyses of off target editing

(A–E) Off-target editing in mouse samples measured by amplicon NGS. Amplicons from Lin^- cells of two untreated and two *in-vivo*-transduced mice were investigated. (A) Top 20 potential off-target sites nominated by CIRCLE-seq. Reads number obtained in CIRCLE-seq and alignment with the on-target sequence are shown. (B) Off-target base editing at the 20 CIRCLE-seq sites. A > G frequencies consistent with ABE8e editing at all adenines within the editable window (positions 3 to 14) are plotted. (C) Top 10 ranked potential off-target sites nominated by *in silico* prediction using Cas-OFFinder. (D) Off-target base editing at the top 10 CIRCLE-seq sites. (E) Indel frequencies around the predicted nicking sites analyzed by Cas-Analyzer. (F–J) Off-target editing in human samples measured by amplicon NGS. Amplicons from $CD34^+$ cells of two SCD patients with or without HDAd-Maka-v3 transduction were sequenced. Panels are similar to (A)–(E) except human samples were used. Data shown are mean with SD where applicable.

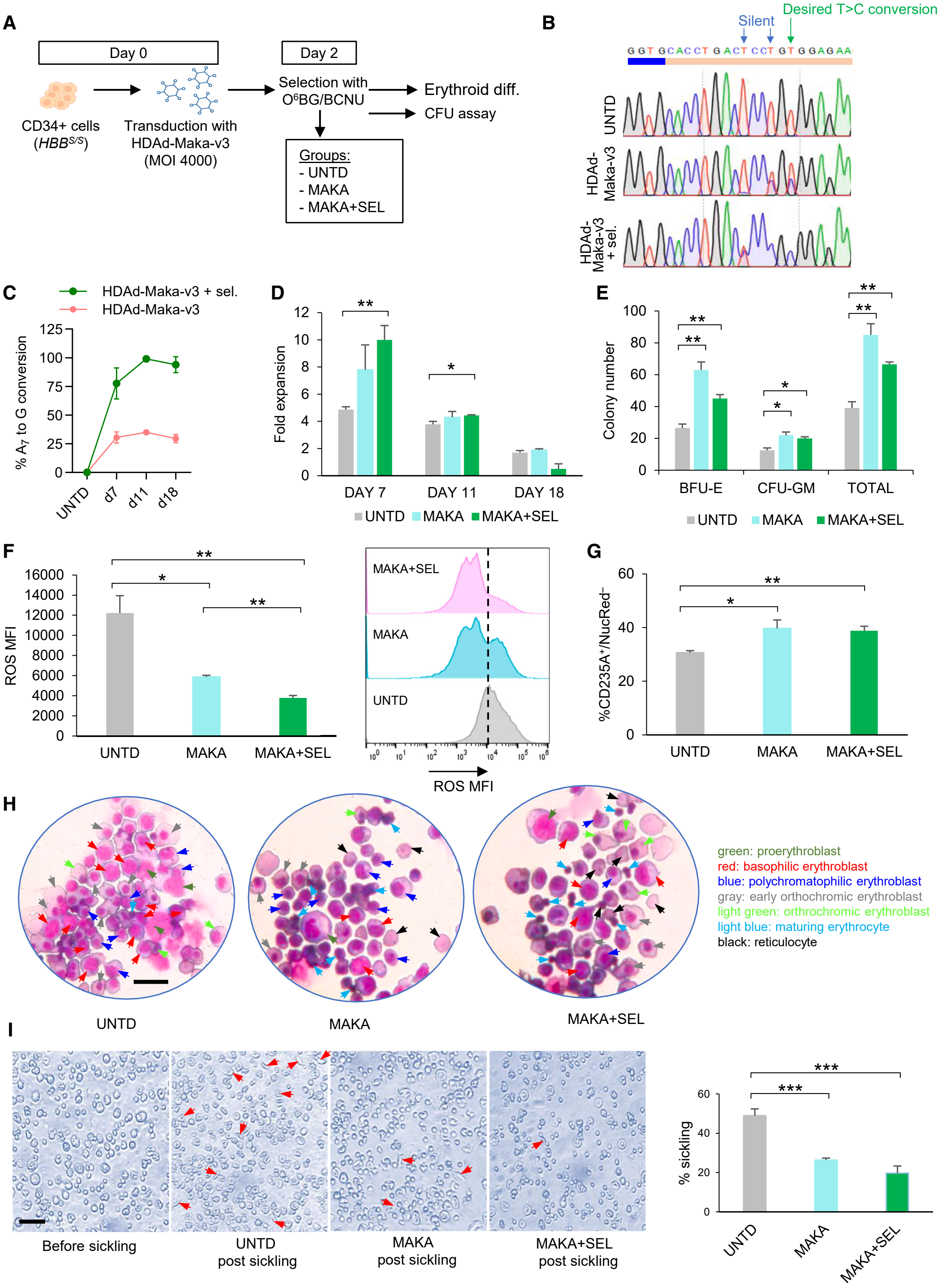

(legend on next page)

CD34+ cells from SCD patients, which achieved ~80% conversion of *HBB^S* to *HBB^G*.[10] This suggests that the HDAd-Maka-v3 vector (without O6BG/BCNU selection) would have therapeutic benefits for *ex vivo* HSC gene therapy.

For *in vivo* HSC transduction studies, HDAd-Maka-v3 was injected intravenously into G-CSF/AMD3100 mobilized CD46/Townes mice. Animals were subjected to O6BG/BCNU selection. At week 16 after *in vivo* transduction, 34% of PBMCs had at least one corrected allele. Notably, for a cure of SCD, editing of only one allele is sufficient. *In vivo* editing in >30% of HbS was converted into HbG, which corrected the disease phenotype, including normalized hematological parameters and reticulocytosis, >50% decreased sickling of erythrocytes, reduced spleen size and hemosiderosis.

Concerns with our *in vivo* approach include the need for O6BG/BCNU selection, which would not be possible in SCD patients. We are working on alternative *in vivo* expansion approaches that do not require chemotherapy drugs, including approaches that involve truncated Epo receptors[28] and epitope editing in combination with antibody-drug-conjugate selection.[29,30] Our HDAd5/35++ vector targets CD46, a receptor that is overexpressed on HSCs, but also present on all other nucleated cells at lower levels. This can result in transduction and editing in non-hematopoietic tissues. While editing of the β-globin gene in these tissues is unlikely to cause side effects, further investigation of edited parenchymal cells could be warranted.

Both base and prime editors do not require DSBs for efficient editing. Both approaches are currently being tested clinically by Beam Therapeutics and Prime Medicine. The following arguments should be considered in the decision on which approach to use for the treatment of SCD: in our studies, ABE8e-NRCH was more effective in installing the corrective mutation in human CD34+ cells than PE5max *in vitro* studies. It remains to be shown whether this is also the case *in vivo*. (We noticed before that *in vitro* editing in primary cells is not necessarily predictive for *in vivo*.) On the other hand, ABE8e-NRCH also mediated bystander (A_9 and A_{12}) editing, which in this case is not critical because these edits are synonymous,[11] but might be critical for other targets within coding regions. For prime editors, no bystander editing was triggered, and on-target edits are >99% of the desired product. Also, although pegRNA design is not always straightforward, the PE machinery inherently minimizes off-target editing. Considering the less stringent requirements for off-targeting by base editors than prime editors, more in-depth and systematic off-target evaluations for ABE8e-NRCH are warranted for future clinical applications. Furthermore, Cas-independent off-target deamination needs to be examined carefully in future studies.[31] It also remains to be shown whether newer prime editors can trigger efficient edits without the need to transiently suppress the cellular DNA repair machinery by dnMLH1.[32]

In summary, our *in vivo* HSC base editing approach for directly repairing the root cause of SCD achieved therapeutically relevant levels of editing and amelioration of disease symptoms in SCD mice. The efficacy of our *in vivo* HSC gene therapy approach was comparable with current marketed *ex vivo* products. If further optimized, our *in vivo* approach could, however, be superior in terms of accessibility, availability, affordability.

MATERIALS AND METHODS

Reagents for *in vivo* transduction and selection

G-CSF (Neupogen) (Amgen, Thousand Oaks, CA), AMD3100 (MilliporeSigma, Burlington, MA), and dexamethasone sodium phosphate (Fresenius Kabi USA, Lake Zurich, IL) were used. O6-Benzylguanine (O6-BG) and carmustine (BCNU) were from MilliporeSigma.

Cloning and production of HDAd-Maka vectors

The oligonucleotides and gBlocks described below were synthesized by Integrated DNA Technologies (Coralville, IA) and listed in Table S5.

HDAd-Maka-v1 and HDAd-Maka-v2

The cloning of version 1 and version 2 vectors involved three steps. Step 1 was construction of shuttle plasmids with all-in-one base editors. The 6.3 kb fragment from pBS-ABE8e-sgHBG#2-miR and the 7.1 kb fragment from pBS-ABE8e-sgHBG#2-miR-v2[33] were PCR amplified using primers #1FR, followed by infusion ligation with the 4.0 kb *ApoI* fragment of pCMV-ABEmax-NRCH (Addgene, no. 136923),[12] generating pBS-ABE8e-NRCH-sgHBG#2-miR (with a PGK promoter driving the editor) and pBS-ABE8e-NRCH-sgHBG#2-miR-v2 (with an EF1a promoter driving the editor), respectively. The sgRNA_Maka gBlock (#2)

Figure 6. *In vitro* studies with CD34+ cells from SCD patients infected with HDAd-Maka
(A) Experimental design. CD34+ cells from SCD patients homozygous for *HBB^{S/S}* ($n = 3$) were isolated by magnetic separation. CD34+ cell purity was on average 57%. Cells were infected with HDAd-Maka-v3 at an MOI of 4,000 vp/cell or left untransduced (UNTD). Two days later, cells were either plated in Methocult medium for progenitor colony assays or subjected to erythroid differentiation with (MAKE+SEL) or without O6BG/BCNU *in vitro* selection (MAKA). (B) Representative DNA chromatogram from Sanger sequencing showing target site T > C conversion (A > G on the complementary strand) in cells of donor 3 with or without selection (day 11). (C) Summarized editing levels at different days after *in vitro* differentiation with or without selection. (D) Expansion of cells during erythroid differentiation. Fold expansion was calculated as the later time point cell number compared with the previous time point cell number. (E) Number of myeloid, erythroid, and total progenitor colonies formed from 2,000 plated CD34+ cells (counted on day 14 of culture). (F) Flow cytometry analysis of reactive oxygen species (ROS) at day 18 of ED/*in vitro* selection. The graph on the right shows the ROS MFIs for donor 3. (G) Percentage of enucleated erythroid cells. (H) Representative cytospins stained with Grünwald/Giemsa. Scale bar, 25 μm. The arrows indicate cells at different stages of erythroid differentiation. (I) Representative images of enucleated erythroid progeny 30 min after sodium metabisulfite treatment. Red arrows indicate sickle forms. Scale bar, 25 μm. Shown on the right is a summary of percentage of sickled cells. Statistical significance was assessed by one-way ANOVA with Šidák's multiple comparisons tests to calculate p values. *$p < 0.05$, **$p < 0.01$, ***$p < 0.001$. Data shown are mean with SD where applicable.

was cloned to the *NdeI-AscI* site of pBS-ABE8e-NRCH-sgHBG#2-miR-v2, generating pBS-ABE8e-NRCH-sgHBB-Maka-v2. Then, the 0.4 kb U6-sgHBB-Maka fragment was amplified from pBS-ABE8e-NRCH-sgHBB-Maka-v2 with primers #3FR and inserted between the two *BamHI* sites of pBS-ABE8e-NRCH-sgHBG#2-miR and pBS-ABE8e-NRCH-sgHBG#2-miR-v2, resulting in pBS-ABE8e-NRCH-sgHBB-Maka-v1 and pBS-ABE8e-NRCH-sgHBB-Maka-v2, respectively. Step 2: the 1.6 kb stuffer DNA amplified from pHCA using #4FR and recombined with *SwaI*-linearized pHCAS2-MCS-FI-PGK-mgmt[5] by Gibson Assembly to form pHCAS3-MCS-FI-PGK-mgmt. Step 3: the v1 shuttle plasmid from step 1 and the pHCAS3-FI-EF1a-mgmt-MCS vector described previously[5] were linearized by *PacI* and joined by infusion to generate pHCA-ABE8e-NRCH-Maka-v1 with a vector genome of 29.7 kb. The v2 shuttle vector from step 1 and the product of step 2 were similarly digested with *PacI* and recombined to form pHCA-ABE8e-NRCH-Maka-v2 (vector genome = 30.6 kb).

HDAd-Test-v1

The cloning is similar to that of pHCA-ABE8e-NRCH-Maka-v1 described above except the sgRNA_Test gBlock (#5) is used.

HDAd-Maka-v3

The version 3 vector was designed with more stringent regulatory elements to suppress transgene expression in the 116 producer cells by adding vaRNAs to the v2 vector. Briefly, the 0.3kb vaRNA_miR183/218_vaRNA fragment was released from pBS-ABE8e-sgHBG#2-miR-v3[33] by *NotI* and cloned into the *NotI* sites of pBS-ABE8e-NRCH-sgHBB-Maka-v2, forming pBS-ABE8e-NRCH-sgHBB-Maka-v3. The 5.8 kb stuffer sequence from pHCA was amplified using #6FR and inserted into the *SwaI* site of pHCAS5-MCS-FI-PGK-mgmt,[5] resulting in pHCAS7-MCS-FI-PGK-mgmt. Next, the products of the above two steps were digested with *PacI* and joined by infusion to generate pHCA-ABE8e-NRCH-Maka-v3 (viral genome = 30.6 kb).

HDAd-Maka-v4 and HDAd-Maka-v5

The v4 and v5 vectors were designed with the TRiP system[17] to suppress the editor expression in 116 cells. The TRiP system leverages bacterial TRAP, which recognizes its target sequence placed upstream of the transgene start codon. The TRAP gene was cloned into the helper virus, which is described below. The TRAP-binding sequence (tbs) is 11 repeats of KAGNN, which was inserted between the EF1a promoter and the editor gene. Specifically, the 1.4 kb sequence amplified from pBS-ABE8e-NRCH-+sgHBB-Maka-v3 using #7FR and the 2.6 kb fragment amplified from the same template using #8FR were joined with *AscI-EcoRV*-digested pBS-ABE8e-NRCH-sgHBB-Maka-v3 by infusion, generating pBS-ABE8e-NRCH-Maka-v4. Here, the tbs sequence was introduced by the #8FR primers. The vaRNA_miR183/218_vaRNA sequence between the two *NotI* sites in pBS-ABE8e-NRCH-Maka-v4 was removed by *NotI* digestion and the vector was self-ligated, resulting in pBS-ABE8e-NRCH-Maka-v5. The two pBS vectors were treated with *PacI* and recombined with *PacI*-digested pHCAS7-MCS-FI-PGK-mgmt, generating pHCA-ABE8e-NRCH-Maka-v4 (viral genome = 30.7 kb) and pHCA-ABE8e-NRCH-Maka-v5 (viral genome = 30.3 kb), respectively.

pAd5/35S++_TRAP helper vector

A codon-optimized TRAP sequence for expression in mammalian cells was synthesized as a gBlock (#9) and inserted into the *EcoRI* sites of pBS-CMV-anti-Cas9, resulting in pBS-CMV-TRAP. The 10.2 kb sequence amplified from pNG163-CMV-antiCas9-A with #10FR was recombined with *AsiSI-BstBI*-digested pNG163-CMV-anti-Cas9-A by Gibson Assembly. The resulting plasmid was digested with *BstBI* and recombined with the 1.5 kb CMV-TRAP-SV40pA fragment amplified from pBS-CMV-TRAP using #11FR, generating pAd5/35S++_TRAP (also referred to as pNG163-TRAP) with a vector genome of 36.8 kb. The helper virus was produced by transfection of HEK293 cells and validated by restriction site analysis.

The Phusion Hot Start II High-Fidelity DNA Polymerase (New England Biolabs, Ipswich, MA) was used in all PCR reactions involved in cloning. Final constructs were screened by several restriction enzymes (*HindIII*, *EcoRI*, *BamHI*, and *PmeI*) and confirmed by Nanopore whole-plasmid sequencing.

The production of HDAd vectors has been described in detail previously.[34] In brief, the corresponding pHCA plasmids were linearized with *PmeI* and transfected into 116 cells. Twenty-four hours later, the cells were infected with the Ad5/35L-Acr helper, which expresses an anti-CRISPR inhibitor and contains a long-shafted 5/35 fiber.[33] The cells were harvested 2 days after infection and subjected to three cycles of freeze-and-thaw to lyse the cells. The lysate was used for further amplification by co-infection of fresh 116 cells with the helper virus. After six rounds of amplification, the capsid was switched to short-shafted 5/35S++ in the last round of amplification using the Ad5/35S++-Acr helper. For the production of HDAd-Maka-v4 and -v5 vectors, the Ad5/35S++_TRAP helper was used. The HDAd viruses were purified by two rounds of ultracentrifugation with cesium chloride density gradients, followed by dialysis in a buffer with 10 mM Tris-HCl (pH 7.5), 10 mM $MgCl_2$, and 10% glycerol. Helper virus contamination levels were found to be <0.05%. Titers were 0.1–3.9 $\times$ 10^{12} vp/mL. The correctness of vector genomes was validated by restriction enzyme digestion and sequencing.

Animal studies

All experiments involving animals were conducted in accordance with the institutional guidelines set forth by the University of Washington. The University of Washington is an Association for the Assessment and Accreditation of Laboratory Animal Care International-accredited research institution and all live animal work conducted at this university is in accordance with the Office of Laboratory Animal Welfare Public Health Assurance policy, USDA Animal Welfare Act and Regulations, the Guide for the Care and Use of Laboratory Animals, and the University of Washington's Institutional Animal Care and Use Committee (IACUC) policies. The studies were approved by the University of Washington IACUC (protocol no. 3108-01). C57Bl/6-based transgenic mice that contained the human CD46 genomic locus and

provide CD46 expression at a level and in a pattern similar to humans (hCD46$^{+/+}$ mice) were described earlier.[35]

SCD mouse model

A Townes male mouse (Hbb$^{tm2(HBG1,HBB*)Tow}$ or hα/hα:β^S/β^S)[36] was purchased from The Jackson Laboratory (JAX stock no. 013071) and bred with human CD46 transgenic female mice. After three rounds of breeding, mice homozygous for *CD46*, *HBB*^{S}, and *HBA* were obtained and used for experiments. All animals involved in the study have the C57BL/6 background. The following primers were used for genotyping: HBB primers: 5′-ATGTCAGAAGCAAATGTGAGGAGCA-3′, 5′-AATTCTGGCTTATCGGAGGCAAG-3′, and 5′-TTGAGCAAT GTGGACAGAGAAGG-3′; HBA primers: 5′-TCCTGCAGGGTGA GGAAGGAAGG-3′, 5′- TCTATGCACATCAATTAGCAGAGGC-3′, and 5′-CCCCAAGGCACTCCAGGGACATAG-3′; and CD46 primers: CD46 forward, 5′-GCCAGTTCATCTTTTGACTCTAT TAA-3′, and reverse, 5′-AATCACAGCAATGACCCAAA-3′.

Ex vivo *HSC gene therapy*

Lineage-negative cells were isolated from total mouse bone marrow cells by MACS using the Lineage Cell Depletion kit from Miltenyi Biotech (Bergisch Gladbach, Germany). Lin$^-$ cells were cultured in IMDM supplemented with 10% FCS, 10% BSA, penicillin/streptomycin, glutamine, 10 ng/mL human thrombopoietin (TPO), 20 ng/mL mouse stem cell factor (SCF), and 20 ng/mL human Flt-3L. After overnight culturing, the cells were transduced with HDAd vectors at an MOI of 500 vp/cell for 24 h, followed by transplantation into recipient mice. Recipients were female C57BL/6J mice, 6–8 weeks old from The Jackson Laboratory. On the day of transplantation, recipient mice were irradiated with 1,000 Rad. Six hours after irradiation cells were injected intravenously at 1×10^6 cells per mouse.

HSC mobilization and in vivo *transduction*

HSCs were mobilized in mice by subcutaneous (s.c.) injections of human recombinant G-CSF (250 μg/kg/mouse/day, 4 days) followed by an s.c. injection of AMD3100 (5 mg/kg) on day 5. In addition, animals received dexamethasone (10 mg/kg, intraperitoneally [i.p.]) 16 and 2 h before virus injection to blunt innate toxicity associated with intravenous HDAd injection. Forty-five minutes after AMD3100, animals were intravenously injected with virus vectors through the retro-orbital plexus (4×10^{10} vp/ mouse).

In vivo *selection*

Selection was started 6 days after transduction. Mice were injected with O^{6}BG (15 mg/kg, i.p.) two times, 30 min apart. One hour after the second injection of O^{6}BG, mice were injected (i.p.) with 5 mg/kg BCNU. At days 19 and 33, two more rounds were performed with BCNU doses of 9 and 10 mg/kg, respectively.

Secondary BM transplantation

BM cells from *ex-vivo*- or *in-vivo*-transduced CD46tg mice were isolated aseptically. Lineage-depleted (Lin$^-$) cells were isolated and transplanted as described above. The secondary recipients were kept for 16 weeks after transplantation for terminal point analyses.

Tissue analysis

Spleen and liver tissue sections of 2.5 μm thickness were fixed in 4% formaldehyde for at least 24 h, dehydrated, and embedded in paraffin. Staining with hematoxylin and eosin was used for histological evaluation of extramedullary hemopoiesis. Hemosiderin was detected in tissue sections by Perls Prussian blue staining. In brief, the tissue sections were treated with a mixture of equal volumes (2%) of potassium ferrocyanide and hydrochloric acid in distilled water and then counterstained with neutral red. The spleen size was assessed as the ratio of spleen weight (mg)/body weight (g).

Blood analyses

Blood samples were collected into EDTA-coated tubes and analyses were performed on a HemaVet 950FS (Drew Scientific, Waterbury, CT). Peripheral blood smears were stained with Giemsa/May-Grünwald (Merck, Darmstadt, Germany) for 5 and 15 min, respectively. Reticulocytes were stained with Brilliant cresyl blue. The investigators who counted the reticulocytes on blood smears have been blinded to the sample group allocation. Only animal numbers appeared on the slides (five slides per animal, five random 1 cm^2 sections). For the sickling assay, 20 mL of blood was mixed with 2% sodium metabisulfite on a microscope slide. Cells were then covered with a coverslip, sealed, and examined under a microscope within 1–4 h at 40× magnification. Images were randomized and blinded. The percentage of sickled cells for each condition was obtained by manually counting the number of sickled cells.

CD34$^+$ cells from SCD patients

CD34$^+$ cells from SCD patients homozygous for *HBB*$^{S/S}$ (n = 3) were immunomagnetically isolated from exchange transfusion blood samples at the George Papanikolaou Hospital. PBMCs were isolated from peripheral blood by Ficoll density gradient centrifugation followed by CD34 positive selection (Miltenyi Biotec). A range between 0.5 and 2.5×10^6 of CD34$^+$ cells were isolated from approximately 320–350 mL of steady-state blood. CD34$^+$ cells were incubated overnight in StemSpan H3000 medium (STEMCELL Technologies, Vancouver, Canada) supplemented with penicillin/streptomycin, Flt3 ligand (Flt3L, 100 ng/mL), TPO (100 ng/mL), and SCF 100 ng/mL). Cytokines and growth factors were from Peprotech (Rocky Hill, NJ). CD34$^+$ cells were transduced with HDAd-Maka-v3 vector at an MOI of 4,000 in low-attachment 12-well plates.

In vitro erythroid differentiation of CD34$^+$ cells with O^{6}BG/BCNU selection

Differentiation of human CD34$^+$ cells into erythroid cells was done based on the protocol developed by Douay et al.[37] In brief, in step 1, cells at a density of 10^4 cells/mL were incubated for 7 days in IMDM supplemented with 5% human plasma, 2 IU/mL heparin, 10 μg/mL insulin, 330 μg/mL transferrin, 1 μM hydrocortisone, 100 ng/mL SCF, 5 ng/mL IL-3, 3 U/mL erythropoietin (Epo), glutamine, and penicillin/streptomycin. In step 2, cells at a density of 1×10^5 cells/mL were incubated for 3 days in IMDM supplemented with 5% human plasma, 2 IU/mL heparin, 10 μg/mL insulin, 330 μg/mL transferrin, 100 ng/mL SCF, 3 U/mL Epo, glutamine,

and penicillin/streptomycin. In step 3, cells at a density of 1×10^6 cells/mL were incubated for 8 days in IMDM supplemented with 5% human plasma, 2 IU/mL heparin, 10 μg/mL insulin, 330 μg/mL transferrin, 3 U/mL Epo, glutamine, and penicillin/streptomycin. For the enrichment of transduced cells, 48 h post-transduction, $CD34^+$ cells were treated with 50 μM O^6BG and 35 μM BCNU. Specifically, cells were incubated with 50 μM fresh O^6BG for 1 h. Without washing, fresh 35 μM BCNU was added for 2.5 h, after which cells were washed and resuspended in fresh medium.

Analysis of ROS levels

Intracellular ROS levels from erythroid precursors were determined using the General Oxidative Stress Indicator CM-H2DCFDA (no. C6827, Life Technologies), according to the manufacturer's instructions. In brief, SCD $CD34^+$ cells were incubated with 10 μM CM-H2DCFDA in PBS at 37°C for 1 h and washed twice with PBS before analysis. Oxidation of the probe can be detected by the increase of fluorescence (GFP) by flow cytometry.

Cytospin slide preparation

Cytospins of $0.3–1.0 \times 10^5$ cells were prepared by cytocentrifugation (ROTOFIX 32, Hettich Zentrifugen) at 500 rpm for 5 min. Cytospins were air dried and then stained with Giemsa for 15 min (Merck) and May-Grünwald for 5 min (Merck) and subjected to imaging analysis.

Sickling assay

In-vitro-differentiated erythroid cells were resuspended at a density of 5×10^5 cells/500 μL in erythroid differentiation medium (as in step 3 of ECD, above). To induce sickling, 500 μL of freshly prepared 2% sodium metabisulfite in PBS were added. Following incubation at room temperature for 30 min, live cell images were captured using a Nikon microscope. The sickling percentage was quantified by manually counting, in 10 optical fields, the number of sickled cells over the total cells, and estimating the average percentage of sickling for each condition.

CFU assay

Lin^- cells were isolated by depletion of lineage-committed cells in BM MNCs using the mouse lineage cell depletion kit (Miltenyi Biotec, San Diego, CA) according to the manufacturer's instructions. CFU assays were performed using ColonyGEL 1202 (Reachbio, Seattle, WA) with mouse complete medium according to the manufacturer's protocol. Colonies were scored 10 days after plating. For human $CD34^+$ cells, ColonyGEL 1102 (Reachbio, Seattle, WA) with human complete medium was used. Colonies derived from human HSPCs were counted on day 14.

Measurement of gene editing by Sanger sequencing and NGS

Genomic segments encompassing the target sites were amplified using primers: forward, 5′- TCGTCGGCAGCGTCAGATGTGTATAAGAGACAGCCATCTATTGCTTACATTTGCTTCTG-3′; reverse, 5′- GTCTCGTGGGCTCGGAGATGTGTATAAGAGACAGAGTTTCTATTGGTCTCCTTAAACCTG-3′ (underlined is partial adaptors for NGS). The amplicons were purified using AMPure XP Beads (Beckman Coulter, Indianapolis, IN), Sanger sequenced with the forward primer shown above and analyzed by using EditR.[38] For NGS, the purified amplicons were submitted to Genewiz (South Plainfield, NJ) for Amplicon-EZ sequencing. Data were aligned to the reference sequence 5′-CCATCTATTGCTTACATTTGCTTCTGACACAACTGTGTTCACTAGCAACCTCAAACAGACACCATGGTGCACCTGACTCCTGAGGAGAAGTCTGCCGTTACTGCCCTGTGGGGCAAGGTGAACGTGGATGAAGTTGGTGGTGAGGCCCTGGGCAGGTTGGTATCAAGGTTACAAGACAGGTTTAAGGAGACCAATAGAAACT-3′ and analyzed using CRISPResso2,[39] a python-based genome editing analysis tool. For quantification of indel frequencies, data were aligned to the above reference sequence using the Cas-Analyzer online tool (http://www.rgenome.net/cas-analyzer/#!),[40] a JavaScript-based implementation for NGS data analysis. A quantification window of 140 bp around the predicted nicking site was used by setting the comparison range as 70 bp.

Flow cytometry

Cells were resuspended at 1×10^6 cells/100 μL in FACS buffer (PBS plus 1% heat-inactivated FBS) and incubated with FcR blocking reagent (Miltenyi Biotech, Auburn CA) for 10 min on ice. Next, the staining antibody solution was added in 100 μL per 10^6 cells and incubated on ice for 30 min in the dark. After incubation, cells were washed once in FACS buffer. For secondary staining, the staining step was repeated with a secondary staining solution. After the wash, cells were resuspended in FACS buffer and analyzed using an LSRII flow cytometer (BD Biosciences, San Jose, CA). Debris was excluded using a forward scatter-area and sideward scatter-area gate. Single cells were then gated using a forward scatter-height and forward scatter-width gate. Flow cytometry data were then analyzed using FlowJo (version 10.0.8, FlowJo, LLC). For analysis of LSK cells, cells were stained with biotin-conjugated lineage detection cocktail (catalog no. 130-092-613) (Miltenyi Biotec, San Diego, CA), antibodies against c-Kit (clone 2B8, catalog no. 12-1171-83) and Sca-1 (clone D7, catalog no. 25-5981-82), followed by secondary staining with APC-conjugated streptavidin (catalog no. 17-4317-82) (eBioscience, San Diego, CA). Other antibodies from eBioscience included anti-mouse CD3-APC (clone 17A2) (catalog no. 17-0032-82), anti-mouse CD19-PE-Cyanine7 (clone eBio1D3) (catalog no. 25-0193-82), anti-mouse Ly-6G/Ly-6C (Gr-1)-PE (clone RB6-8C5) (catalog no. 12-5931-82, and anti-human CD235-FITC (clone HIR2) (catalog no. 11-9987-82). Anti-mouse TER-119-APC (clone TER-119) (catalog no. 116211) was from BioLegend (San Diego, CA). Anti-human CD45-APC (clone 5B1) was from Miltenyi Biotec (catalog no. 130-108-020). Anti-human CD34-PE (clone 563) was from STEMCELL Technologies. Anti-human CD90-Brilliant Violet 605 (clone 5E10) (catalog no. 562685) and anti-human CD38-PerCP/Cy5.5 (clone HIT2) (catalog no. 561106) was from BD Biosciences. Anti-human CD235a-PE (clone JC159) (catalog no. 1P-784-T100) and anti-human CD36-APC (clone CB38) (catalog no. 1A-648-T100) were from ExBIO. The nuclear stain was performed using NucRed Live 647 (catalog no. R37106) from Thermo Fisher Scientific.

Detection of hemoglobin subunits by mass spectrometry

RBCs in 10 μL of whole-blood samples were lysed by adding 190 μL ultrapure water, vortexing vigorously for 10 s, and incubating at room temperature for 5 min. The lysates were cleared up by spinning at 13,000 × *g* for 10 min at room temperature. Twenty microliters of supernatants was collected, further diluted in 180 μL of ultrapure water, vortexed, and spun again as described above. Thirty microliters of the light reddish supernatants containing hemoglobin subunits was analyzed on an AB Sciex TripleTOF 5600 mass spectrometer equipped with an electrospray ionization DuoSpray source. The system was operated with Analyst software, version 1.6 (ABSciex, Framingham, MA). Chromatographic separation of analytes was achieved using a PLRP-S reversed-phase column (50 mm length; 2.1 mm inner diameter; 100 Å pore size; 3 μm particle size) (Agilent Technologies, Santa Clara, CA). A water/acetonitrile gradient with 0.1% formic acid was used to separate analytes. Chromatograms of hemoglobin subunits were generated by using PeakView software, version 2.2 (ABSciex). *m*/*z* values ranging from 600 to 1,600 were reconstructed for globin peaks. Peak areas of β-globin variants were used for calculating their percentages of total β-globins.

Off-target analysis by CIRCLE-seq

Circularization for *in vitro* reporting of cleavage effects by sequencing (CIRCLE-seq) was performed and analyzed as described previously.[10,23] In brief, genomic DNA isolated from bone marrow cells of CD46/Townes was sheared with a Covaris S2 instrument to an average length of 300 bp. The fragmented DNA was end repaired, A tailed, and ligated to a uracil-containing stem-loop adaptor, using a KAPA HTP Library Preparation Kit, PCR Free (KAPA Biosystems). Adaptor-ligated DNA was treated with Lambda Exonuclease (NEB) and *E. coli* Exonuclease I (NEB), and then with USER enzyme (NEB) and T4 polynucleotide kinase (NEB). Intramolecular circularization of the DNA was performed with T4 DNA ligase (NEB) and residual linear DNA was degraded by Plasmid-Safe ATP-dependent DNase (Lucigen). *In vitro* cleavage reactions were performed with 250 ng of Plasmid-Safe-treated circularized DNA, 90 nM of Cas9-NRCH protein, Cas9 nuclease buffer (NEB), and 90 nM of synthetic chemically modified sgRNA (BioSpring) in a 100 μL volume. Cleaved products were A tailed, ligated with a hairpin adaptor (NEB), treated with USER enzyme (NEB), and amplified by PCR with barcoded universal primers NEBNext Multiplex Oligos for Illumina (NEB), using Kapa HiFi Polymerase (KAPA Biosystems). Libraries were sequenced with 150 bp paired-end reads on an Illumina MiSeq instrument. CIRCLE-seq data analyses were performed using open-source CIRCLE-seq analysis software and default recommended parameters (https://github.com/tsailabSJ/circleseq). The top 20 off-target sites with highest reads numbers nominated by CIRCLE-seq were further analyzed by amplicon deep sequencing (see measurement of gene editing by Sanger sequencing and NGS). Primers used for PCR and reference sequences used for analysis were listed in Table S1. For off-target editing in humans, CIRCLE-seq was performed previously.[10] The top 10 sites showed off-target activity with RNP and mRNA electroporation were analyzed in the current study (Tables S3 and S4). Amplicons from $CD34^+$ cells of two SCD patients with or without HDAd-Maka-v3 transduction were sequenced as above.

Off-target analysis by Cas-OFFinder

Potential off-target sites in whole mouse and human genome were also computationally nominated by Cas-OFFinder.[24] Candidates with mismatches ≤3 bp to the on-target sequence are listed in Tables S2 and S4. The top 10 sites were experimentally investigated by amplicon NGS as described above. PCR primers and reference sequences used for analysis were listed in the two tables.

Statistical analyses

Statistical significance was calculated by appropriate statistical tests as described in the figure legends. Statistical analysis was computed on GraphPad Prism version 9.0.0 (GraphPad Software, La Jolla, CA). $p < 0.05$ was considered as statistically different.

DATA AND CODE AVAILABILITY

NGS data have been deposited to the NCBI Sequence Read Archive (SRA) with the project code PRJNA1121293. This SRA submission will be released upon publication.

ACKNOWLEDGMENTS

The study was supported by NIH grants R01HL128288 (to A.L.), R01HL141781 to A.L.), by a grant from Ensoma Bio (to A.L. and H.-P.K.), and by a grant from the Bill and Melinda Gates Foundation: INV-017692 (to A.L.). C.L. was supported by an award from the University of Washington Department of Medicine chair. D.R.L. was supported by NIH grants UG3AI150551, U01AI142756, R35GM118062, RM1HG009490, and R01HL136135; the Bill and Melinda Gates Foundation; and HHMI. Under the grant conditions of the BMGF, a Creative Commons Attribution 4.0 Generic License has already been assigned to the Author Accepted Manuscript version that might arise from this submission. We thank Linda Y. Mamiya and Dale Whittington for technical support for isoelectric focusing, high-performance liquid chromatography, and mass spectrometry.

AUTHOR CONTRIBUTIONS

C.L. provided the conceptual framework for the study. C.L. and E.Y. designed the experiments. C.L., A.G., K.P., A.K.A., L.H., S.G., M.G., E.V., and G.A.N. performed the experiments. D.R.L. and H.-P.K. provided critical comments on the manuscript. C.L. and A.L. wrote the manuscript.

DECLARATION OF INTERESTS

A.L. and H.-P.K. are academic co-founders of Ensoma Therapeutics. H.-P.K. is a paid advisor for Ensoma. D.R.L. is a consultant and co-founder of Prime Medicine, Beam Therapeutics, Pairwise Plants, Chroma Medicine, companies that use genome or epigenome engineering agents.

SUPPLEMENTAL INFORMATION

Supplemental information can be found online at https://doi.org/10.1016/j.ymthe.2024.10.018.

REFERENCES

1. Kanter, J., Walters, M.C., Krishnamurti, L., Mapara, M.Y., Kwiatkowski, J.L., Rifkin-Zenenberg, S., Aygun, B., Kasow, K.A., Pierciey, F.J., Jr., Bonner, M., et al. (2022). Biologic and Clinical Efficacy of LentiGlobin for Sickle Cell Disease. N. Engl. J. Med. *386*, 617–628. https://doi.org/10.1056/NEJMoa2117175.
2. Grimley, M., Asnani, M., Kent, M., Shrestha, A., Felker, S., Lutzko, C., Arumugam, P., Witting, S., Knight-Madden, K., Niss, O., et al. (2022). High Anti-Sickling Potency of a Gamma Globin in the Phase 1/2 MOMENTUM Study of ARU-1801 Gene Therapy and Reduced Intensity Conditioning for Sickle Cell Disease. Hemasphere *23*, 1335.

3. Frangoul, H., Altshuler, D., Cappellini, M.D., Chen, Y.S., Domm, J., Eustace, B.K., Foell, J., de la Fuente, J., Grupp, S., Handgretinger, R., et al. (2021). CRISPR-Cas9 Gene Editing for Sickle Cell Disease and beta-Thalassemia. N. Engl. J. Med. *384*, 252–260. https://doi.org/10.1056/NEJMoa2031054.

4. Esrick, E.B., Lehmann, L.E., Biffi, A., Achebe, M., Brendel, C., Ciuculescu, M.F., Daley, H., MacKinnon, B., Morris, E., Federico, A., et al. (2021). Post-Transcriptional Genetic Silencing of BCL11A to Treat Sickle Cell Disease. N. Engl. J. Med. *384*, 205–215. https://doi.org/10.1056/NEJMoa2029392.

5. Li, C., Georgakopoulou, A., Newby, G.A., Chen, P.J., Everette, K.A., Paschoudi, K., Vlachaki, E., Gil, S., Anderson, A.K., Koob, T., et al. (2023). In vivo HSC prime editing rescues sickle cell disease in a mouse model. Blood *141*, 2085–2099. https://doi.org/10.1182/blood.2022018252.

6. Chen, P.J., Hussmann, J.A., Yan, J., Knipping, F., Ravisankar, P., Chen, P.F., Chen, C., Nelson, J.W., Newby, G.A., Sahin, M., et al. (2021). Enhanced prime editing systems by manipulating cellular determinants of editing outcomes. Cell *184*, 5635–5652.e29. https://doi.org/10.1016/j.cell.2021.09.018.

7. Sangkitporn, S., Rerkamnuaychoke, B., Sangkitporn, S., Mitrakul, C., and Sutivigit, Y. (2002). Hb G Makassar (beta 6:Glu-Ala) in a Thai family. J. Med. Assoc. *85*, 577–582.

8. Blackwell, R.Q., Oemijati, S., Pribadi, W., Weng, M.I., and Liu, C.S. (1970). Hemoglobin G Makassar: beta-6 Glu leads to Ala. Biochim. Biophys. Acta *214*, 396–401.

9. Viprakasit, V., Wiriyasateinkul, A., Sattayasevana, B., Miles, K.L., and Laosombat, V. (2002). Hb G-Makassar [beta6(A3)Glu–>Ala; codon 6 (GAG–>GCG)]: molecular characterization, clinical, and hematological effects. Hemoglobin *26*, 245–253. https://doi.org/10.1081/hem-120015028.

10. Newby, G.A., Yen, J.S., Woodard, K.J., Mayuranathan, T., Lazzarotto, C.R., Li, Y., Sheppard-Tillman, H., Porter, S.N., Yao, Y., Mayberry, K., et al. (2021). Base editing of haematopoietic stem cells rescues sickle cell disease in mice. Nature *595*, 295–302. https://doi.org/10.1038/s41586-021-03609-w.

11. Chu, S.H., Packer, M., Rees, H., Lam, D., Yu, Y., Marshall, J., Cheng, L.I., Lam, D., Olins, J., Ran, F.A., et al. (2021). Rationally Designed Base Editors for Precise Editing of the Sickle Cell Disease Mutation. CRISPR J. *4*, 169–177. https://doi.org/10.1089/crispr.2020.0144.

12. Miller, S.M., Wang, T., Randolph, P.B., Arbab, M., Shen, M.W., Huang, T.P., Matuszek, Z., Newby, G.A., Rees, H.A., and Liu, D.R. (2020). Continuous evolution of SpCas9 variants compatible with non-G PAMs. Nat. Biotechnol. *38*, 471–481. https://doi.org/10.1038/s41587-020-0412-8.

13. Li, C., Anderson, A.K., Ruminski, P.G., Rettig, M.P., Karpova, D., Kiem, H.P., DiPersio, J.F., and Lieber, A. (2024). A simplified, G-CSF-free procedure allows for in vivo HSC gene therapy of sickle cell disease in a mouse model. Blood Adv. *18*, 4089. https://doi.org/10.1182/bloodadvances.2024012757.

14. Li, C., Goncalves, K.A., Raskó, T., Pande, A., Gil, S., Liu, Z., Izsvák, Z., Papayannopoulou, T., Davis, J.C., Kiem, H.P., and Lieber, A. (2021). Single-dose MGTA-145/plerixafor leads to efficient mobilization and in vivo transduction of HSCs with thalassemia correction in mice. Blood Adv. *5*, 1239–1249. https://doi.org/10.1182/bloodadvances.2020003714.

15. Li, C., Georgakopoulou, A., Newby, G.A., Everette, K.A., Nizamis, E., Paschoudi, K., Vlachaki, E., Gil, S., Anderson, A.K., Koob, T., et al. (2022). In vivo base editing by a single i.v. vector injection for treatment of hemoglobinopathies. JCI Insight *7*, e162939. https://doi.org/10.1172/jci.insight.162939.

16. Palmer, D.J., Turner, D.L., and Ng, P. (2019). Production of CRISPR/Cas9-Mediated Self-Cleaving Helper-Dependent Adenoviruses. Mol. Ther. Methods Clin. Dev. *13*, 432–439. https://doi.org/10.1016/j.omtm.2019.04.003.

17. Maunder, H.E., Wright, J., Kolli, B.R., Vieira, C.R., Mkandawire, T.T., Tatoris, S., Kennedy, V., Iqball, S., Devarajan, G., Ellis, S., et al. (2017). Enhancing titres of therapeutic viral vectors using the transgene repression in vector production (TRiP) system. Nat. Commun. *8*, 14834. https://doi.org/10.1038/ncomms14834.

18. Merino, E., Babitzke, P., and Yanofsky, C. (1995). trp RNA-binding attenuation protein (TRAP)-trp leader RNA interactions mediate translational as well as transcriptional regulation of the Bacillus subtilis trp operon. J. Bacteriol. *177*, 6362–6370. https://doi.org/10.1128/jb.177.22.6362-6370.1995.

19. Li, C., Psatha, N., Gil, S., Wang, H., Papayannopoulou, T., and Lieber, A. (2018). HDAd5/35(++) Adenovirus Vector Expressing Anti-CRISPR Peptides Decreases CRISPR/Cas9 Toxicity in Human Hematopoietic Stem Cells. Mol. Ther. Methods Clin. Dev. *9*, 390–401. https://doi.org/10.1016/j.omtm.2018.04.008.

20. Richter, M., Saydaminova, K., Yumul, R., Krishnan, R., Liu, J., Nagy, E.E., Singh, M., Izsvák, Z., Cattaneo, R., Uckert, W., et al. (2016). *In vivo* transduction of primitive mobilized hematopoietic stem cells after intravenous injection of integrating adenovirus vectors. Blood *128*, 2206–2217. https://doi.org/10.1182/blood-2016-04-711580.

21. Wang, H., Richter, M., Psatha, N., Li, C., Kim, J., Liu, J., Ehrhardt, A., Nilsson, S.K., Cao, B., Palmer, D., et al. (2018). A Combined In Vivo HSC Transduction/Selection Approach Results in Efficient and Stable Gene Expression in Peripheral Blood Cells in Mice. Molecular therapy. Methods Clin. Dev. *8*, 52–64. https://doi.org/10.1016/j.omtm.2017.11.004.

22. Mohamad, A.S., Hamzah, R., Selvaratnam, V., Yegapan, S., and Sathar, J. (2018). Human hemoglobin G-Makassar variant masquerading as sickle cell anemia. Hematol. Rep. *10*, 7210. https://doi.org/10.4081/hr.2018.7210.

23. Tsai, S.Q., Nguyen, N.T., Malagon-Lopez, J., Topkar, V.V., Aryee, M.J., and Joung, J.K. (2017). CIRCLE-seq: a highly sensitive in vitro screen for genome-wide CRISPR-Cas9 nuclease off-targets. Nat. Methods *14*, 607–614. https://doi.org/10.1038/nmeth.4278.

24. Bae, S., Park, J., and Kim, J.S. (2014). Cas-OFFinder: a fast and versatile algorithm that searches for potential off-target sites of Cas9 RNA-guided endonucleases. Bioinformatics *30*, 1473–1475. https://doi.org/10.1093/bioinformatics/btu048.

25. Li, C., Wang, H., Gil, S., Germond, A., Fountain, C., Baldessari, A., Kim, J., Liu, Z., Georgakopoulou, A., Radtke, S., et al. (2022). Safe and efficient in vivo hematopoietic stem cell transduction in nonhuman primates using HDAd5/35++ vectors. Molecular therapy. Mol. Ther. Methods Clin. Dev. *24*, 127–141. https://doi.org/10.1016/j.omtm.2021.12.003.

26. Frangoul, H., Locatelli, F., Sharma, A., Bhatia, M., Mapara, M., Molinari, L., Wall, D., Liem, R.I., Telfer, P., Shah, A.J., et al. (2024). Exagamglogene Autotemcel for Severe Sickle Cell Disease. N. Engl. J. Med. *390*, 1649–1662. https://doi.org/10.1056/NEJMoa2309676.

27. Kanter, J., Thompson, A.A., Pierciey, F.J., Jr., Hsieh, M., Uchida, N., Leboulch, P., Schmidt, M., Bonner, M., Guo, R., Miller, A., et al. (2023). Lovo-cel gene therapy for sickle cell disease: Treatment process evolution and outcomes in the initial groups of the HGB-206 study. Am. J. Hematol. *98*, 11–22. https://doi.org/10.1002/ajh.26741.

28. Luna, S.E., Camarena, J., Hampton, J.P., Majeti, K.R., Charlesworth, C.T., Soupene, E., Selvaraj, S., Jia, K., Sheehan, V.A., Cromer, M.K., and Porteus, M.H. (2024). Enhancement of erythropoietic output by Cas9-mediated insertion of a natural variant in haematopoietic stem and progenitor cells. Nat. Biomed. Eng. *1*, 1. https://doi.org/10.1038/s41551-024-01222-6.

29. Wellhausen, N., O'Connell, R.P., Lesch, S., Engel, N.W., Rennels, A.K., Gonzales, D., Herbst, F., Young, R.M., Garcia, K.C., Weiner, D., et al. (2023). Epitope base editing CD45 in hematopoietic cells enables universal blood cancer immune therapy. Sci. Transl. Med. *15*, eadi1145. https://doi.org/10.1126/scitranslmed.adi1145.

30. Garaude, S., Marone, R., Lepore, R., Devaux, A., Beerlage, A., Seyres, D., Dell' Aglio, A., Juskevicius, D., Zuin, J., Burgold, T., et al. (2024). Selective haematological cancer eradication with preserved haematopoiesis. Nature *630*, 728–735. https://doi.org/10.1038/s41586-024-07456-3.

31. Zhou, C., Sun, Y., Yan, R., Liu, Y., Zuo, E., Gu, C., Han, L., Wei, Y., Hu, X., Zeng, R., et al. (2019). Off-target RNA mutation induced by DNA base editing and its elimination by mutagenesis. Nature *571*, 275–278. https://doi.org/10.1038/s41586-019-1314-0.

32. Doman, J.L., Pandey, S., Neugebauer, M.E., An, M., Davis, J.R., Randolph, P.B., McElroy, A., Gao, X.D., Raguram, A., Richter, M.F., et al. (2023). Phage-assisted evolution and protein engineering yield compact, efficient prime editors. Cell *186*, 3983–4002.e26. https://doi.org/10.1016/j.cell.2023.07.039.

33. Li, C., Georgakopoulou, A., Newby, G.A., Everette, K.A., Nizamis, E., Paschoudi, K., Vlachaki, E., Gil, S., Anderson, A.K., Koob, T., et al. (2022). In vivo base editing by a single intravenous vector injection for treatment of hemoglobinopathies. JCI Insight *7*, e162939.

34. Palmer, D.J., and Ng, P. (2008). Methods for the production of helper-dependent adenoviral vectors. Methods Mol. Biol. *433*, 33–53. https://doi.org/10.1007/978-1-59745-237-3_3.

35. Kemper, C., Leung, M., Stephensen, C.B., Pinkert, C.A., Liszewski, M.K., Cattaneo, R., and Atkinson, J.P. (2001). Membrane cofactor protein (MCP; CD46) expression in transgenic mice. Clin. Exp. Immunol. *124*, 180–189.

36. Ryan, T.M., Ciavatta, D.J., and Townes, T.M. (1997). Knockout-transgenic mouse model of sickle cell disease. Science *278*, 873–876. https://doi.org/10.1126/science.278.5339.873.

37. Douay, L., and Giarratana, M.C. (2009). Ex vivo generation of human red blood cells: a new advance in stem cell engineering. Methods Mol. Biol. *482*, 127–140. https://doi.org/10.1007/978-1-59745-060-7_8.

38. Kluesner, M.G., Nedveck, D.A., Lahr, W.S., Garbe, J.R., Abrahante, J.E., Webber, B.R., and Moriarity, B.S. (2018). EditR: A Method to Quantify Base Editing from Sanger Sequencing. CRISPR J. *1*, 239–250. https://doi.org/10.1089/crispr.2018.0014.

39. Clement, K., Rees, H., Canver, M.C., Gehrke, J.M., Farouni, R., Hsu, J.Y., Cole, M.A., Liu, D.R., Joung, J.K., Bauer, D.E., and Pinello, L. (2019). CRISPResso2 provides accurate and rapid genome editing sequence analysis. Nat. Biotechnol. *37*, 224–226. https://doi.org/10.1038/s41587-019-0032-3.

40. Park, J., Lim, K., Kim, J.S., and Bae, S. (2017). Cas-analyzer: an online tool for assessing genome editing results using NGS data. Bioinformatics *33*, 286–288. https://doi.org/10.1093/bioinformatics/btw561.

YMTHE, Volume *32*

Supplemental Information

Introducing a hemoglobin G-Makassar variant in HSCs by *in vivo* base editing treats sickle cell disease in mice

Chang Li, Aphrodite Georgakopoulou, Kiriaki Paschoudi, Anna K. Anderson, Lishan Huang, Sucheol Gil, Maria Giannaki, Efthymia Vlachaki, Gregory A. Newby, David R. Liu, Evangelia Yannaki, Hans-Peter Kiem, and André Lieber

Supplemental methods

Hemoglobin HPLC. Ion-exchange HPLC was performed to measure hemoglobins in hemolysates. We used the VARIANT II TURBO System (Bio-Rad, Hercules, CA) at the Red Cell Disorders Lab of the Harborview Medical Center by following the manufacturer's instructions. Hemolysates were prepared from EDTA blood samples collected from sCD46/Townes mice by using the Wash/Diluent Solution provided for the instrument.

Hemoglobin electrophoresis by isoelectric focusing (IEF). Detection of hemoglobin variants by IEF was performed at the Red Cell Disorders Lab of the Harborview Medical Center. The RESOLVE™ Hemoglobin kit from PerkinElmer (Waltham, MA) was used. Briefly, whole blood samples were diluted in RESOLVE™ solution to release the hemoglobin proteins in red blood cells, vortexed, and spun at 14,000×g for 4 min at room temperature. 4.5 µL of supernatants was loaded onto the agarose IEF gel provided in the kit. Electrophoretic separation was performed at 10°C with a Multiphor II Electrophoresis Unit (GE Healthcare, Chicago, IL), using a power supply at a voltage limit of 1500 V and a current and power limit of 2.75 mA/cm and 1.2 W/cm gel, respectively. The gel was fixed, stained with Coomassie blue R250, destained for clear background, and air dried for imaging analysis.

Propidium iodide staining for cell cycle analysis. 2-5 × 10^5 cells were fixed by adding 200 µl of 70% ethanol, then incubated at 4°C for 30 minutes. Following a second wash with 2 ml PBS + 2% BSA and centrifugation at 300 × g for 5 minutes, the cells were stained with 0.5 ml propidium iodide (PI)/RNase solution (Immunostep, Salamanca, Spain), incubated at room temperature for 15 minutes, and analyzed by flow cytometry to assess the DNA content.

Annexin/7-AAD staining for apoptosis analysis. The PE Annexin V Apoptosis Detection Kit I from BD Pharmingen (catalog # 559763) was used. 1 × 10^5 cells were washed twice with cold PBS and resuspended in 1X Binding Buffer provided in the kit at a concentration of 1 × 10^6 cells/ml. The cells were stained with PE Annexin V (5 µl) and 7-AAD (5 µl), incubated at room temperature for 15 minutes in the dark. After incubation, 400 µl of 1X Binding Buffer was added. Flow cytometry analysis was performed within 1 hour.

Table S1. Potential off-target sites in mouse genome identified by CIRCLE-Seq. Genomic DNA from CD46/Townes naive mice was cleaved with recombinant Cas9-NRCH plus the guide sequence sgRNA_Maka for CIRCLE-Seq. A total of 2372 sites were nominated. The top 20 sites, highlighted in grey, were further analyzed by amplicon deep sequencing. Primers used for PCR and amplicon reference sequences used for analyses were listed.

Table S2. Top-scored potential off-target sites for guide sequence sgRNA_Maka in mouse genome (mm10). Candidates were computationally predicted by Cas-OFFinder and those with mismatches =<3 nucleotides were listed. The top 10 sites highlighted in grey were amplified and further analyzed by amplicon deep sequencing. The column "Overlapping with CIRCLE-Seq candidates?" shows whether those sites are overlapping with candidates nominated by CIRCLE-Seq. If yes, the corresponding ID in Table S1 was listed.

Table S3. Top 10 off-target sites in human genome identified by CIRCLE-seq and amplicon sequencing. The sites were reported previously by Newby GA et al (PMID: 34079130). The ranking were based on off-target editing rates measured by amplicon NGS.

Table S4. Top-scored off-target sites in human genome nominated by Cas-OFFinder. Candidates were computationally predicted by Cas-OFFinder and those with mismatches =<3 nucleotides were listed. The top 10 sites highlighted in grey were amplified and further analyzed by amplicon deep sequencing. The column "Overlapping with CIRCLE-Seq candidates?" shows whether those sites are overlapping with candidates nominated by CIRCLE-Seq reported previously by Newby GA et al (PMID 34079130). If yes, the corresponding ID is listed.

Table S5. Oligo and gBlock sequences used for cloning.

ID	Name	Sequence (5' to 3')	Notes
#1FR	pBS to NRCH_F	cgacggcagcgAATTCGAGCCCAAGAAGAAGAG	For cloning pBS-ABE8e-NRCH-sgHBG#2-miR and pBS-ABE8e-NRCH-sgHBG#2-miR-v2
	pBS to NRCH_R	ccagcaccttgaatttcttgctgggcaccttgtactcgtcggtgatcacggcc<br>cagcccacagagttggtgccgatggTcaggccgatgc	
#2	sgRNA_Maka	AAATGGACTATCATATGCTTACCGTAACTTGAAAGTATTTCGATTTCTTGGCT<br>TTATATATCTTGTGGAAAGGACGAAACACCGTTCTCCAC*A*GGAGTCAGGTGGT<br>TTTAGAGCTAGAAATAGCAAGTTAAAATAAGGCTAGTCCGTTATCAACTTGAA<br>AAAGTGGCACCGAGTCGGTGCTTTTTTTCCGCGGTGGGCGCGCCGGATCCTAA	For cloning pBS-ABE8e-NRCH-sgHBB-Maka-v1
#3FR	U6gRNA-F	CGTTAATTAAGGATCCGAGGGCCTATTTCCCATGATTCC	For cloning pBS-ABE8e-NRCH-sgHBB-Maka-v1 and pBS-ABE8e-NRCH-sgHBB-Maka-v2
	U6gRNA-R	AAATCGATTAGGATCCGGCGCGCCCACCGCGGAAAAAAAGCACCGAC	
#4FR	S2 to S3_F	atatctatgacatttaaataggggattacttgaac	For cloning pHCAS3-MCS-FI-PGK-mgmt
	S2 to S3_R	ttacgattaatatttaaatATATGCCCTCATCAGCCAAATC	
#5	sgRNA_Test	AAATGGACTATCATATGCTTACCGTAACTTGAAAGTATTTCGATTTCTTGGCT<br>TTATATATCTTGTGGAAAGGACGAAACACCGTTCTCCTC*A*GGAGTCAGGTGGT<br>TTTAGAGCTAGAAATAGCAAGTTAAAATAAGGCTAGTCCGTTATCAACTTGAA<br>AAAGTGGCACCGAGTCGGTGCTTTTTTTCCGCGGTGGGCGCGCCGGATCCTAA	For cloning pBS-ABE8e-NRCH-sgHBB-Test-v1
#6FR	S5 to S7_Swal_F	gattaagtggcatttaaatGAATTCTTTTAAAGCTTCCCCC	For cloning pHCAS7-MCS-FI-PGK-mgmt
	S5 to S7_Swal_R	agtaatcccctatttaaatGTCATAGATATATTTGGGGAG	
#7FR	tbs to v4_F1	tttttccgcggtgggcgcgccggatcctaatcgaTTTGAGTAATTCATACA	For cloning pHCAS7-MCS-FI-PGK-mgmt
	tbs to v4_R1	actcgagatctgagtccggtagcgctagcggatctgaccCTCACGACACCTGA<br>AATGGA	
#8FR	tbs to v4_F2	actcagatctcgagtttagcggagtggagaagagcggagccgagcctagcaga<br>gacgagaagagctacagccaccatgaaacggaca	For cloning pBS-ABE8e-sgHBG#2-miR-v2
	tbs to v4_R2	CAGGGTCAGCACGATATCTTCC	
#9	TRAP-pA gBlock	agctggctaggaattcgctgccaccatgaaccagaagcactctagcgacttcg<br>tggtcattaaggccgtggaagatggcgtgaacgtgatcggcctgacaagagga<br>acagacaccaagttccaccattccgagaagctggacaagggcgaagtgatcat<br>cgcccagtttaccgagcacaccagcgctatcaaggtgcggggcgaggccctga<br>tccagaccgcctacggcgaaatgaaaagcgagaaaaagtgagaattcaaaccc<br>gctgatcagcagacatgataagatacattgatgagtttggacaaaccacaact<br>agaatgcagtgaaaaaaatgctttatttgtgaaatttgtgatgctattgcttt<br>atttgtaaccattataagctgcaataaacaagttaacaacaacaattgcattc<br>attttatgtttcaggttcagggggaggtgtgggaggttttttaaagcaagtaa<br>aacctctacaaatgtggtaaaactagtagcggtgggctctatggcttctgagg<br>cggaaagaaccagctggggctctagggggtatccccacgcgccctgtagcggc<br>gcattaagcgcggcgggtgtggtggttacgcgcagcgtgaccgctacacttgc<br>cagcgccctagcgcccgctcctttcgctttcttcccttcctttctcgccacgt<br>tcgccggctttccccgtcaagctctaaatcgggggctccctttagggttccga<br>tttagtgctttacggcacctcgaccccaaaaaacttgattagggtgatggttc<br>acaaatgctagagcttatcgataccgtcgacctcgagggggggcccgatcgaa<br>ttcctgcagcccg	Blue, TRAP; Green, SV40pA; Underlined, EcoRI
#10FR	NG163_del_F	aaaagtttctttgcgatc	For cloning pNG163-CMV-TRAP
	NG163_del_R	tagacgtttaattcgaacaattagctaaatgaattcgag	
#11FR	TRAP to NG_F	ttagctaattgttcgaaCATGAAGAATCTGCTTAGGGTTAG	For cloning pNG163-CMV-TRAP
	TRAP to NG_R	tagacgtttaattcgaaTCGATAAGCTCTAGCATTTGTG	

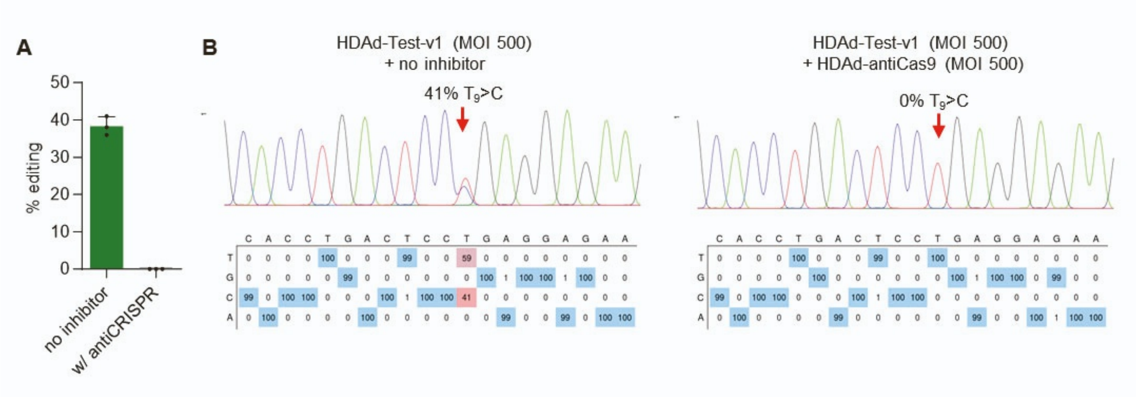

Figure S1. Blockade of base editing by anti-Cas9.

HEK293 cells were infected by HDAd-Test-v1 alone at a MOI of 500 vp/cell or together with HDAd-antiCas9 at an equal MOI. Editing was measured 4 days after vector transduction. A) Percentage of A_9 > G conversion at position 9 (T>C conversion in the complementary strand) measured by Sanger sequencing. Data shown are mean with SD of three biological replicates. B) Representative Sanger sequencing data analyzed by EditR showing base frequencies at the target region.

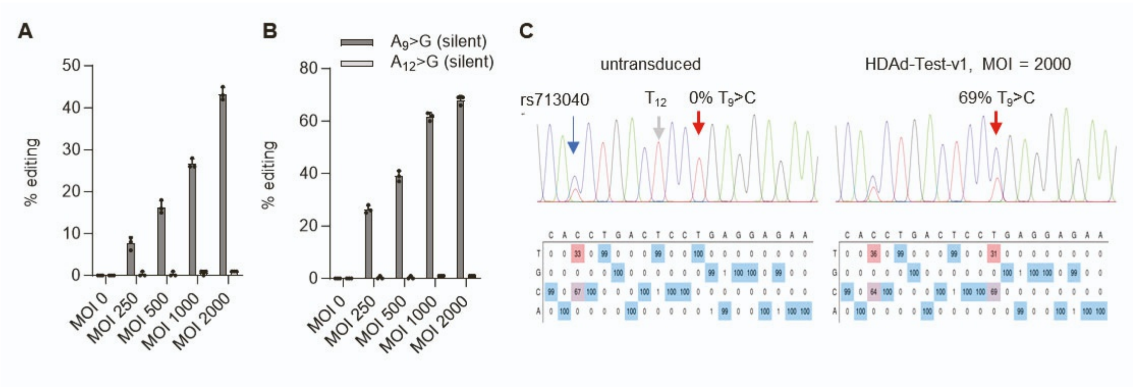

Figure S2. Editing mediated by HDAd-Test-v1.

HEK293 (**A**) or K562 (**B**) cells were infected with HDAd-Test-v1 at indicated MOIs. Base conversions at A_9 and A_{12} were measured 4 days after vector transduction by Sanger sequencing. Data shown are mean with SD of three biological replicates. **C)** Representative Sanger sequencing data of K562 cells (MOI = 2000 vp/cells) analyzed by EditR showing base frequencies at the target bases. Note that the T_9 > C conversion corresponds to A_9 > G in the complementary strand. The blue arrow points to a single-nucleotide polymorphism located in one of the three chromosome 11s of K562.

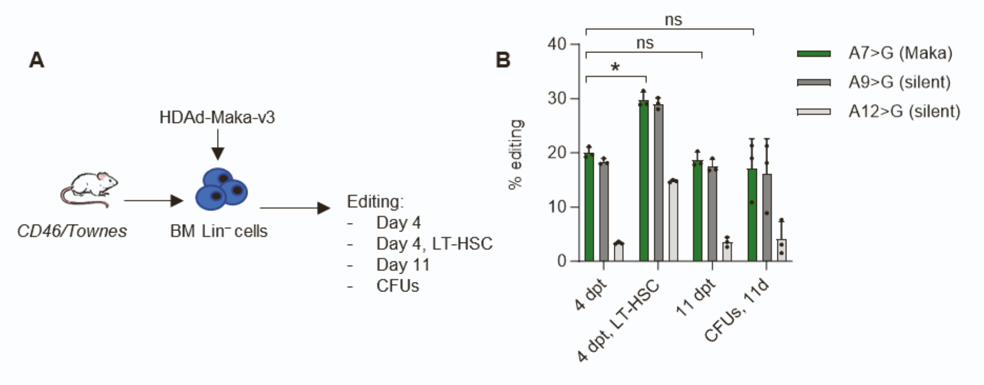

Figure S3. Editing *in vitro* after HDAd-Maka-v3 transduction and extended culture.

A) Schematic of the experiment. Bone marrow lineage-negative (BM Lin^{-}) cells from CD46/Townes mice were transduced with HDAd-Maka-v3 (MOI = 500 vp/cell). Cells were either kept in liquid culture or plated in MethoCult media for CFUs. Editing were measured in bulk BM Lin^{-} cells or sorted long-term HSCs (LT-HSCs, $CD150^{+}CD48^{-}Lin^{-}Sca\text{-}1^{+}c\text{-}Kit^{+}$) at indicated time points after transduction. Pooled CFUs were harvested 10 days after plating for editing analysis. **B)** Target site editing measured by NGS. Each dot represents an individual mouse. Statistical significance was analyzed by one-way ANOVA with Šidák's multiple comparisons tests to compute p-values. dpt, days post-transduction. *, $p<0.05$; ns, not significant.

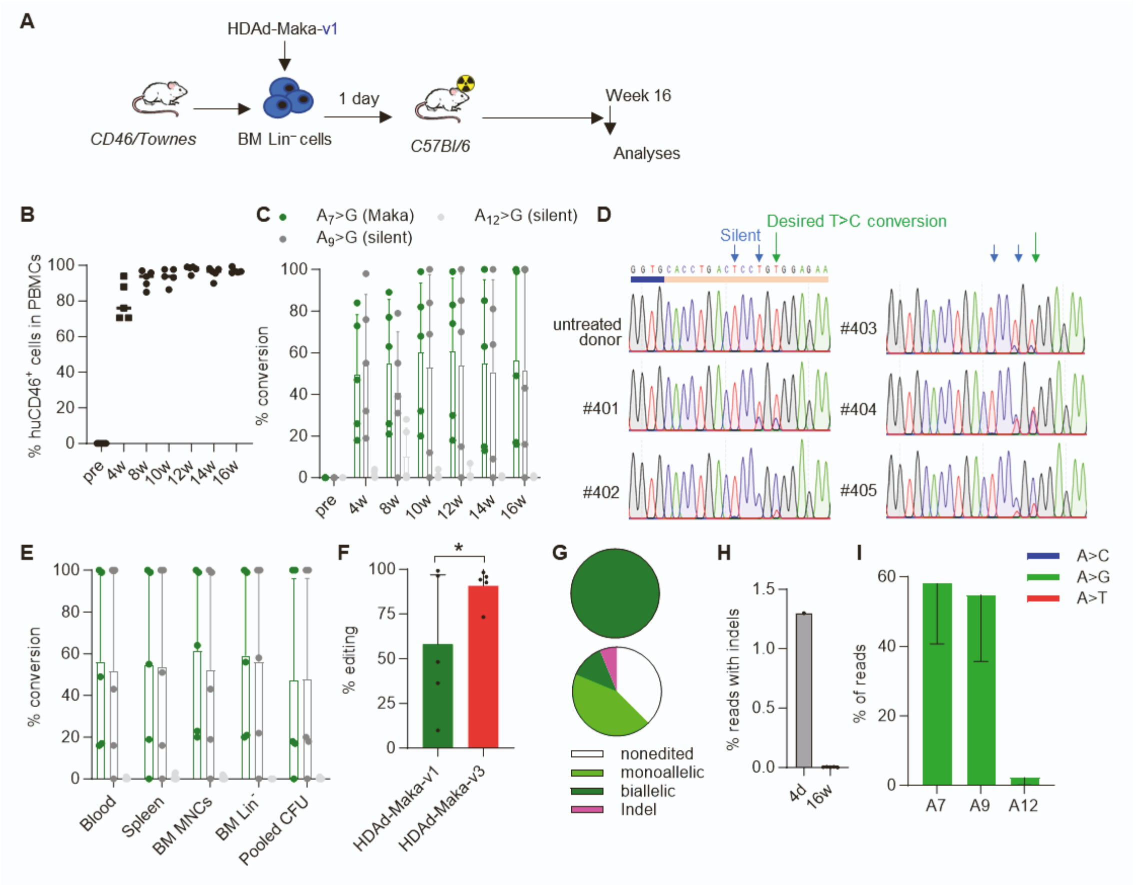

Figure S4. Test HDAd-Maka-v1 in SCD mice by *ex vivo* HSC transduction.

A) Schematic of the experiment. Bone marrow lineage-negative (BM Lin⁻) cells isolated from CD46/Townes mice were transduced with HDAd-Maka-v1 (MOI = 500 vp/cell). Cells were then either cultured for 3 days or transplanted into lethally irradiated C57Bl/6 mice 24 hours after transduction and the transplanted mice were followed for 16 weeks. **B)** Engraftment measured by flow cytometry of human CD46 expression in PBMCs. **C)** Editing in PBMCs at different time points after transplantation. A_7 > G conversion generates the Makassar variant while bystander edits at position A_9 and A_{12} are silent. **D)** Sequencing chromatograms of an untreated donor and week 4 samples after transplantation. The desired T > C conversion (A_7 > G in the complementary strand) was indicated by the green arrow. Bystander edits were indicated by blue arrows. The reference sequence is in 5' to 3' orientation. The blue-orange bar below the reference sequence illustrates the PAM-spacer mapping to the complementary strand. **E)** Editing at the target site in various tissues of primary mice at necropsy. **F)** Comparison of editing rates at the target site in week 16 primary mice. **G)** Allelic analysis in progenitor colonies derived from week 16 BM Lin⁻ cells (n=15) of primary recipients. Two mice were analyzed. **H-I)** Indels (**H**) and base substitution frequencies (I) at target site of Lin⁻ cells at necropsy measured by NGS. A > G conversions were predominant while A > C and A > T conversions were minimal. For **B, C, E, and F**, each dot represents an individual mouse.

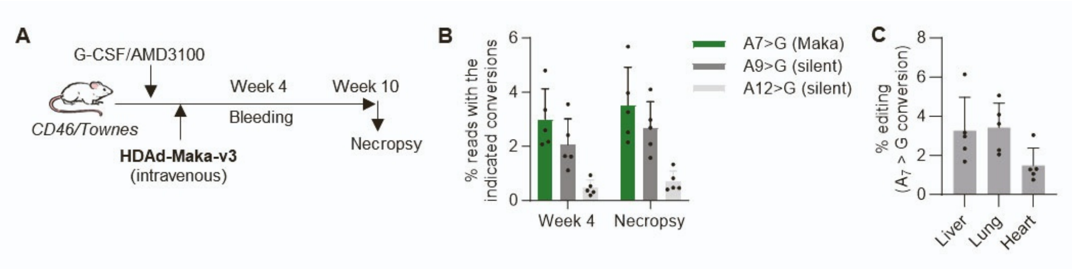

Figure S5. ***In vivo*** **HSC base editing with HDAd-Maka-v3 without** ***in vivo*** **selection.**

A) Schematic of the experiment. SCD mice (n = 5) were *in vivo* transduced with HDAd-Maka-v3 similarly as done for Figure 3. The mice were bled at week 4 and euthanized 10 weeks after in vivo transduction.

B) Editing in PBMCs (week 4) and bone marrow lineage-negative (BM Lin$^-$) cells (necropsy) were measured by NGS. **C)** Editing in tissues at necropsy measured by NGS. Each dot represents a mouse.

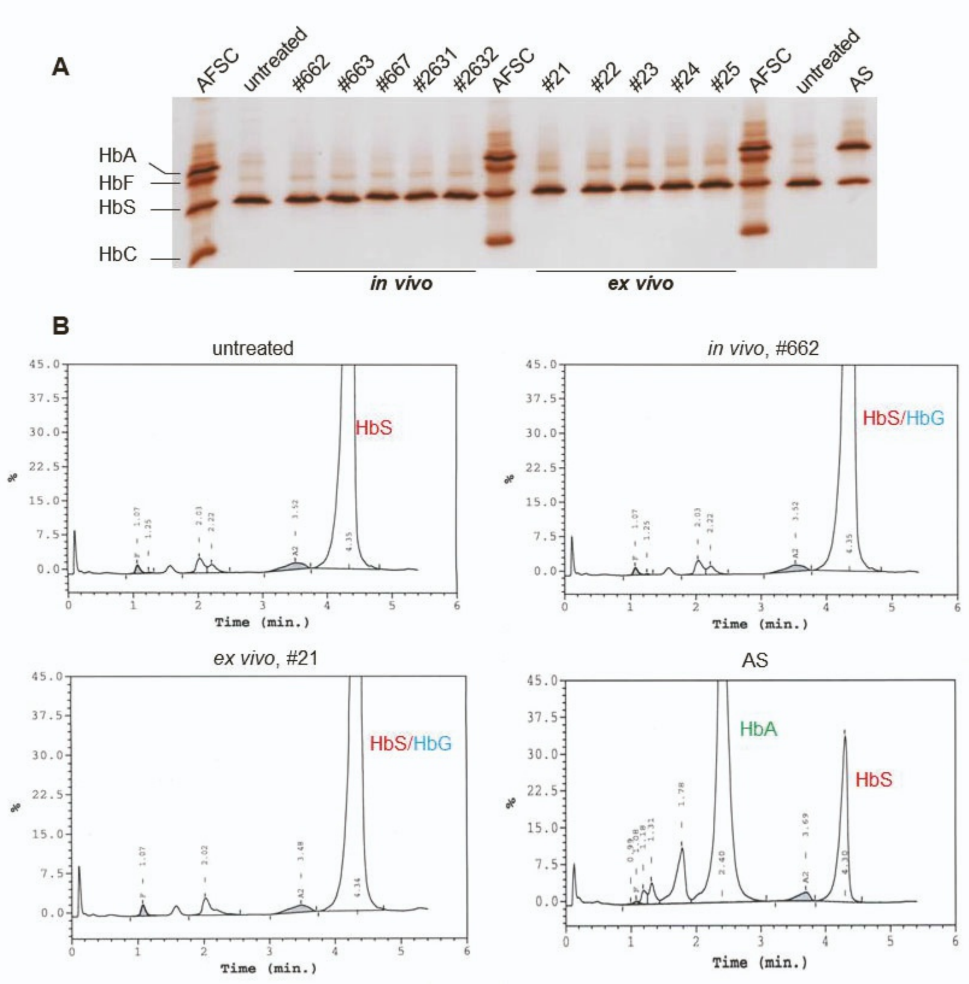

Figure S6. The G-Makassar variant overlaps with HbS in high-performance liquid chromatography (HPLC) or isoelectric focusing (IEF).

A) Analysis of hemoglobin variants by isoelectric focusing electrophoresis. Each lane represents one mouse (ear tag number labeled) or AFSC controls. Bands in AFSC controls indicating four different hemoglobin variants are labeled. **B)** Analysis of hemoglobin variants by ion-exchange high-performance liquid chromatography (HPLC). Representative chromatograms are shown. Week 16 samples of mice *in vivo* transduced with HDAd-Maka-v3 ("*in vivo*") or primary recipients transplanted with HDAd-Maka-v3-transduced CD46/Townes Lin⁻ cells ("*ex vivo*"). AS, blood sample of a control Townes mouse with the HBB^A/HBB^S genotype.

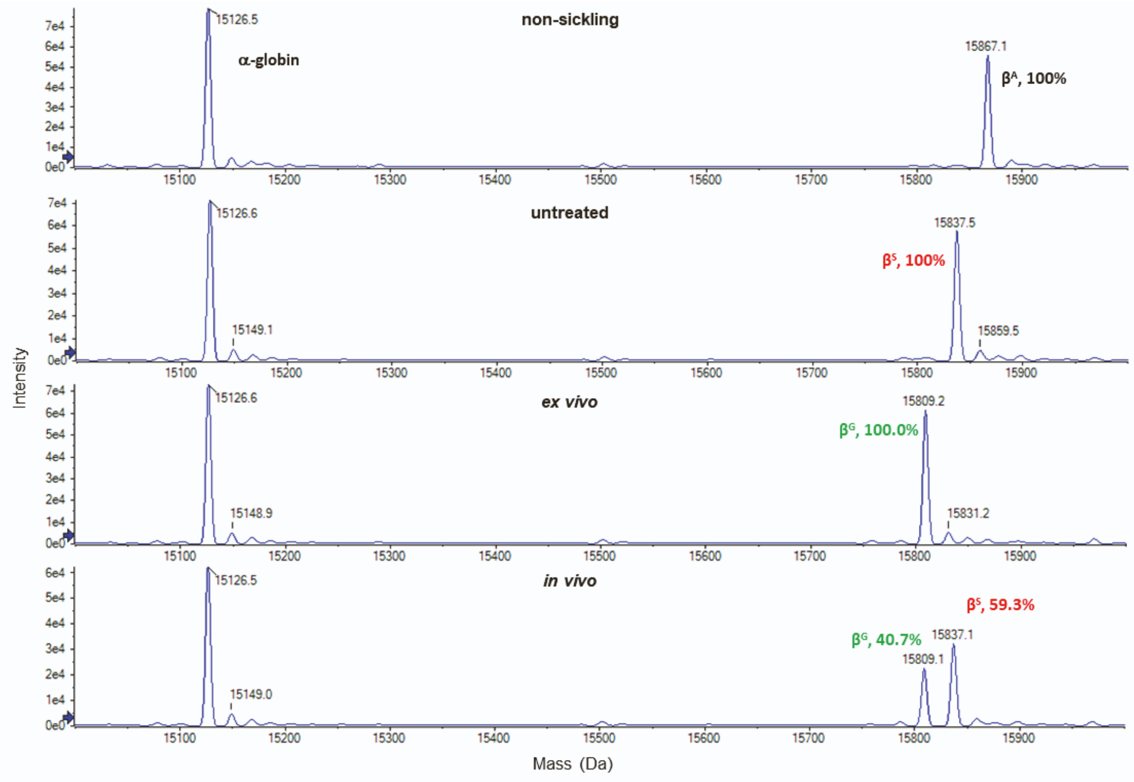

Figure S7. Complete spectrum of chromatograms showing the separation of hemoglobin subunits by mass spectrometry.

The peaks for α, β^A, β^S, β^G chains are labeled.

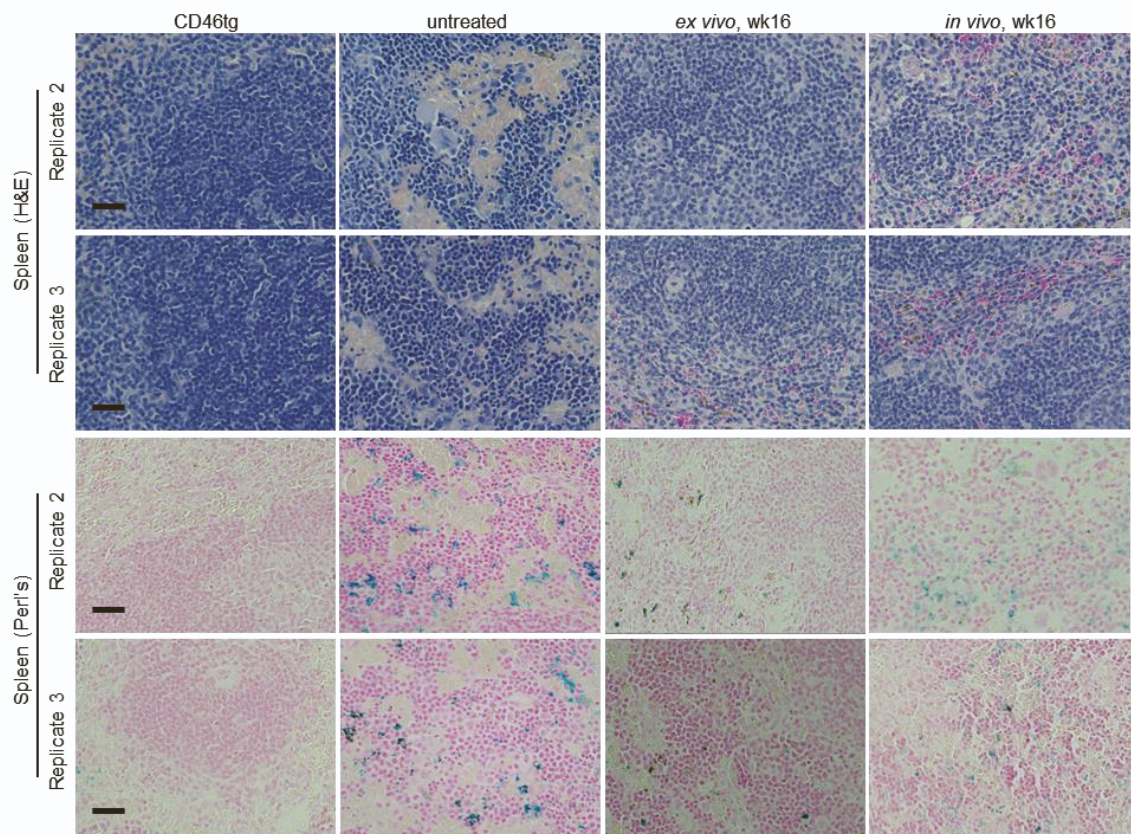

Figure S8. Two more replicates of spleen sections as shown in Figure 4F.

The sections were stained with H&E (two top panels) or Perls' Prussian blue (two bottom panels). The scale bars are 200 µm.

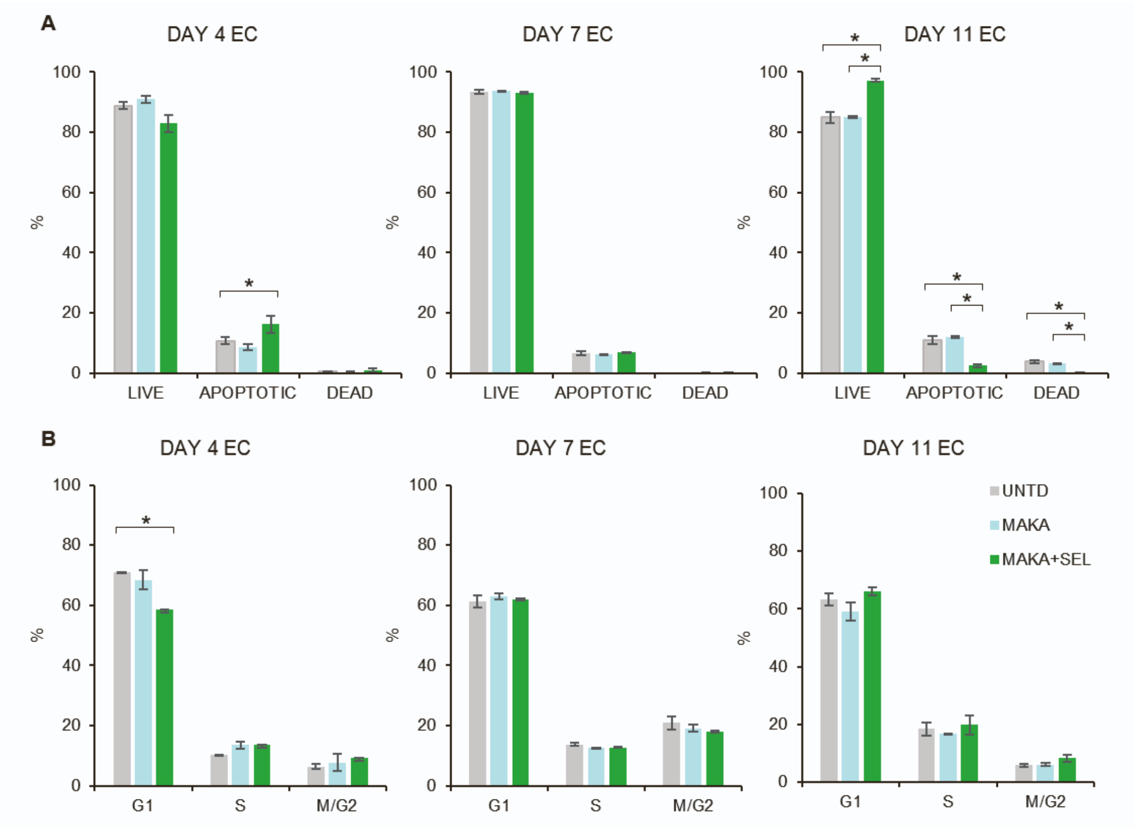

Figure S9. Cell cycle and apoptosis analyses during erythroid differentiation culture.

CD34[+] cells from SCD patients were treated as described in the Fig. 6 legend and cells in different stages of erythroid cells (EC) differentiation culture were analyzed. **A**) Cell cycle was measured by propidium iodide (PI) staining. **B**) Apoptosis was measured by Annexin and 7-AAD staining. Statistical significance was computed by one-way ANOVA with Šidák's multiple comparisons tests to calculate p-values. *, $p<0.05$. UNTD, untransduced. MAKA, transduced with HDAd-Maka-v3. MAKA+SEL, transduced with HDAd-Maka-v3 followed by O^6BG/BCNU selection.
